# Demographic and environmental drivers of metagenomic viral diversity in vampire bats

**DOI:** 10.1111/mec.15250

**Published:** 2019-10-23

**Authors:** Laura M. Bergner, Richard J. Orton, Julio A. Benavides, Daniel J. Becker, Carlos Tello, Roman Biek, Daniel G. Streicker

**Affiliations:** ^1^ Institute of Biodiversity, Animal Health and Comparative Medicine College of Medical, Veterinary and Life Sciences University of Glasgow Glasgow UK; ^2^ MRC–University of Glasgow Centre for Virus Research Glasgow UK; ^3^ Departamento de Ecología Facultad de Ciencias de la Vida Universidad Andrés Bello Santiago Chile; ^4^ Centro de Investigación para la Sustentabilidad Facultad de Ciencias de la Vida Universidad Andrés Bello Santiago Chile; ^5^ Odum School of Ecology University of Georgia Athens GA USA; ^6^ Center for the Ecology of Infectious Diseases University of Georgia Athens GA USA; ^7^ Department of Biology Indiana University Bloomington IN USA; ^8^ Association for the Conservation and Development of Natural Resources Lima Peru; ^9^ Yunkawasi Lima Peru

**Keywords:** Chiroptera, community assembly, demography, *Desmodus rotundus*, elevational gradient, infectious diseases, population structure, shotgun metagenomics, virome, wildlife disease

## Abstract

Viruses infect all forms of life and play critical roles as agents of disease, drivers of biochemical cycles and sources of genetic diversity for their hosts. Our understanding of viral diversity derives primarily from comparisons among host species, precluding insight into how intraspecific variation in host ecology affects viral communities or how predictable viral communities are across populations. Here we test spatial, demographic and environmental hypotheses explaining viral richness and community composition across populations of common vampire bats, which occur in diverse habitats of North, Central and South America. We demonstrate marked variation in viral communities that was not consistently predicted by a null model of declining community similarity with increasing spatial or genetic distances separating populations. We also find no evidence that larger bat colonies host greater viral diversity. Instead, viral diversity follows an elevational gradient, is enriched by juvenile‐biased age structure, and declines with local anthropogenic food resources as measured by livestock density. Our results establish the value of linking the modern influx of metagenomic sequence data with comparative ecology, reveal that snapshot views of viral diversity are unlikely to be representative at the species level, and affirm existing ecological theories that link host ecology not only to single pathogen dynamics but also to viral communities.

## Abstract 2

https://doi.org/10.1111/mec.15306

## 1 INTRODUCTION

Viruses occur across all environments that support life, where they play crucial roles in ecosystem function and in the health of their hosts (Manrique et al., 2016; Suttle, 2005). Additionally, viruses have played foundational evolutionary roles by introducing novel diversity into host genomes (Feschotte & Gilbert, 2012) which has, for instance, contributed to the development of mammalian placentas (Chuong, 2013; Mi et al., 2000) and enhanced antiviral defence through host domestication of viral proteins (Arnaud et al., 2007; Yan, Buckler‐White, Wollenberg, & Kozak, 2009). Our understanding of the factors that influence viral diversity in nature currently derives primarily from comparisons of viral communities between different host species. These studies have revealed that viral communities are shaped by host traits, including body mass and geographical range overlap with other species (Luis et al., 2013; Olival et al., 2017; Turmelle & Olival, 2009). At the population level, however (i.e., among populations of the same host species), the degree to which viral communities are structured by variation in demography and environmental heterogeneity, stochastic forces or host evolutionary history remains unclear.

Understanding whether spatial and ecological variation among populations can explain viral diversity is an important first step if we ultimately aim to forecast changes in viral communities in response to environmental changes or disease control interventions (Anthony et al., 2015; Johnson, Roode, & Fenton, 2015). Such community‐level changes may be important due to direct, immunological or ecological interactions between viruses (DaPalma, Doonan, Trager, & Kasman, 2010; Díaz‐Muñoz, 2017; Rohani, Green, Mantilla‐Beniers, & Grenfell, 2003). Under a null model, geographically proximate populations would be expected to have more similar viral communities than distant populations. Alternatively, viral communities could be structured by demographic or environmental factors that are largely independent of distance; these include established determinants of single viral species such as host population size, age structure and cross‐species transmission (Keeling & Rohani, 2008). Large‐scale biogeographical factors such as latitudinal gradients influence the diversity of human pathogens (Guernier, Hochberg, & Guégan, 2004), and analogous factors such as elevational gradients may create nonlinear relationships between viral communities and distance over smaller spatial scales, but these relationships have never been tested. Finally, viral communities may be so strongly influenced by stochastic factors that prediction is intractable in most natural systems using either spatial proximity or ecological variables that can be realistically measured in the field.

With the exception of studies focused on humans (Manrique et al., 2016; Minot et al., 2011; Robles‐Sikisaka et al., 2013), comparisons of viral communities across populations or time points remain rare (Anthony et al., 2015; Wille et al., 2018). A key barrier has been our inability to characterize unbiased viral communities. The predominance of single pathogen diagnostics, financial constraints and a priori uncertainty of which pathogens are likely to be detected have created ascertainment biases toward certain viral groups (Young & Olival, 2016). Metagenomic sequencing is an alternative data source that enables the simultaneous characterization of entire viral communities (Edwards & Rohwer, 2005) but is rarely applied across multiple populations of the same host species. Applying metagenomic sequencing to spatially replicated data sets therefore offers a new opportunity to understand how host ecology and biogeography influence viral diversity, particularly if applied to host species that occur across habitat types or environmental gradients.

The common vampire bat (*Desmodus rotundus*) is a widespread species that inhabits tropical rainforests, high‐elevation mountains and coastal deserts across North, Central and South America and thrives in both native and anthropogenically transformed ecosystems (Martins, Templeton, Pavan, Kohlbach, & Morgante, 2009; Quintana & Pacheco, 2007). This habitat generalism means that vampire bats live in colonies with extensive variation in climate, anthropogenic food resources and presence of other bat species. Colonies also vary in demographic traits that might impact viral community structure, such as population size, age structure and sex ratio (Delpietro, Marchevsky, & Simonetti, 1992; Delpietro, Russo, Carter, Lord, & Delpietro, 2017; Greenhall, Joermann, Schmidt, & Seidel, 1983; Streicker et al., 2012) (Table 1). As with most broadly distributed species, populations vary in their relative geographical proximity and genetic relatedness (Martins et al., 2009; Streicker et al., 2016), providing an opportunity to test the null expectation that viral community similarity declines with increasing geographical and genetic distance between populations (Nekola & White, 1999). Finally, viral diversity of vampire bats is a major concern for human and animal health, as these bats routinely feed on the blood of livestock and to a lesser degree humans, and are the primary reservoir of rabies virus in the region (Greenhall et al., 1983; Johnson, Aréchiga‐Ceballos, & Aguilar‐Setien, 2014; Voigt & Kelm, 2006). The burden of vampire bat rabies is significant, with an estimated cost of US$50 million per year in livestock losses (Belotto, Leanes, Schneider, Tamayo, & Correa, 2005) and with an estimated force of infection of up to 960 rabies cases per 100,000 inhabitants in a hypothetical outbreak (Schneider et al., 1996). Demographic and ecological factors driving viral dispersal in vampire bats have been investigated specifically for rabies virus (Streicker et al., 2016; de Thoisy et al., 2016; Torres et al., 2014) but such questions have not yet been examined at the level of viral communities.

**Table 1:** Ecological factors that could influence viral richness and community composition in vampire bats

Hypothesized factor	Tested variable (colony‐level)	Predicted effect on richness	References
Host genetic distance	*F*_IS_	↓ Isolation (reduced gene flow) decreases viral invasion of new colonies and increases extinction	Cross, Lloyd‐Smith, Johnson, and Getz (2005), Turmelle and Olival (2009), Plowright et al. (2011)
Colony size	*N*_c_	↑ Greater viral persistence within colonies and increased viral encounters externally	Bartlett (1957); Nunn, Altizer, Jones, and Sechrest (2003); Lloyd‐Smith et al. (2005); Lindenfors et al. (2007)
Age structure	Proportion adults	↑↓ Adults accumulate more chronic infections over a lifetime but juveniles play key roles in viral dynamics due to heightened susceptibility	Lo, Morand, and Galzin (1998); Nunn et al. (2003); Cross et al. (2005); Benavides et al. (2011); Poirotte et al. (2015)
Sex ratio	Proportion males	↑ Males are more susceptible to infections due to behaviour and physiology (testosterone)	Zuk and McKean (1996); Poulin (1996); Reimchen and Nosil (2001); Negro, Caudron, Dubois, Delahaut, and Gemmell (2010)
Local climate	PC1 of mean temperature, temperature range and yearly rainfall PCA (Figure S1)	↓ Climates with higher productivity have higher viral diversity; sites with high temperature and rainfall tend to be negative for PC1	Guernier et al., 2004; Nunn, Altizer, Sechrest, and Cunningham (2005); Dunn, Davies, Harris, and Gavin (2010)
Elevation	Elevation	↓ Diversity tends to decrease with elevation	Lomolino (2001); Wang et al. (2011)
Location	Longitude (latitude excluded due to correlation with climate)	↑↓ Location effects encompass a number of factors so predictions are unclear	Anthony et al. (2015); Chow and Suttle (2015)
Other hosts	Presence/absence of other bat species	↑ Higher diversity of other species provides more opportunities for cross‐species viral transmission	Davies and Pedersen (2008); Krasnov et al. (2010); Huang, Bininda‐Emonds, Stephens, Gittleman, and Altizer (2013); Luis et al. (2015)
Anthropogenic food resources	Livestock density (10‐km radius)	↑↓ Lower prey diversity in areas of high livestock density might reduce diversity of viruses in bats	Gillespie, Chapman, and Greiner (2005); Gay et al. (2014); Bernardo et al. (2018); Becker et al. (2018)

Note

This table presents general hypotheses, specifically tested variables, predicted effect on viral richness, and examples from the literature.

We applied metagenomic sequencing to characterize communities of viruses found in saliva and faecal samples (termed saliva and faecal viruses) across 24 populations of vampire bats spanning the Coast, Andes and Amazon of Peru. By combining viral diversity measures with host genetics and ecological field data, we (a) quantified the extent of variation in viral diversity across populations of a single host species; (b) evaluated whether colonies in the same biogeographical regions or those that are closely connected, either spatially or genetically, exhibit more similar viral communities; and (c) tested how population‐level demographic and environmental factors (Table 1) influence colony‐level viral diversity.

## 2 MATERIALS AND METHODS

### 2.1 Authorizations

Bat sampling methods were approved by the Research Ethics Committee of the University of Glasgow School of Medical, Veterinary and Life Sciences (Ref081/15), the University of Georgia Animal Care and Use Committee (A2014 04‐016‐Y3‐A5), and the Peruvian Government (RD‐009‐2015‐SERFOR‐DGGSPFFS, RD‐264‐2015‐SERFOR‐DGGSPFFS, RD‐142‐2015‐SERFOR‐DGGSPFFS, RD‐054‐2016‐SERFOR‐DGGSPFFS).

### 2.2 Field sampling and demographic variables

**Figure 1:**
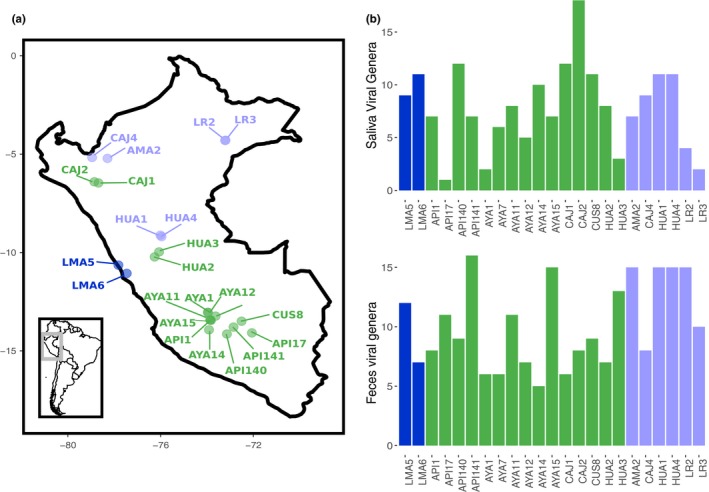
Vampire bat colonies and viral richness summary. (a) Vampire bat colonies in Peru where metagenomic and host genetic samples were taken and (b) summary of viral genera richness in saliva and faeces at each colony. Individual colonies in (a) are represented as coloured points with colony names corresponding to bars in (b). Colours of sites in (a) and bars in (b) correspond to ecoregions within Peru (blue, Coast; green, Andes; purple, Amazon)

Bats were captured at 24 colonies in eight departments across Peru (Figure 1a) between 2013 and 2016 (Table S1) using hand nets within roosts during the day, using mist nets and harp traps to capture bats exiting to forage at night, or by placing nets around nearby livestock when roosts were inaccessible. Nocturnal captures lasted from ~18:00 to 06:00 hr, and nets were checked every 30 min. A combination of one to three mist nets and one harp trap were used depending on the size and number of roost exits identified. Roosts included natural (caves, trees) and anthropogenic structures (abandoned houses, tunnels, mines) which had been previously identified by local farmers and offices of the Ministry of Agriculture of Peru.

Individual bat measures including age and sex were summarized at the colony level to explore effects on viral diversity. Age was determined by examining the level of fusion of the phalangeal epiphyses (Anthony, 1988), which differentiated bats into four age classes. Reproductive status was assessed by the presence of enlarged, scrotal testes in males and pregnancy or lactation in females (Streicker et al., 2012). Capture records from 2016 (the year most samples were collected) were used to calculate colony‐level age and sex ratios, and these ratios were correlated with the age and sex ratios analysed within sequencing pools (Pearson correlation; males *r* = .85, *p* < .001; adults *r* = .84; *p* < .001; see below). Historical capture records (2011–2016) established the presence or absence of other bat species at each roost site (Table S2); however, bat diversity was not calculated as the effort in identifying other species was inconsistent across sites and years.

Bats were given uniquely numbered wing bands (3.5‐mm incoloy, Porzana Inc.) to identify recaptures. To examine the effect of colony size on viral diversity, census population size (*N*_c_) was estimated from mark–recapture data for each colony, but data were unavailable from five sites (two were exclusively sampled during the day using hand nets and three were sampled by placing nets around nearby livestock precluding comparable mark–recapture analyses; Appendix S1; Table S2). Exploratory analyses of the remaining 19 sites indicated *N*_c_ was not significantly correlated with viral richness in faeces (Pearson correlation; all viruses *r* = .15, *p* = .54; vertebrate‐infecting viruses *r* = .43, *p* = .08) or saliva (Pearson correlation; all viruses *r* = −.05, *p* = .84; vertebrate‐infecting viruses *r* = .10, *p* = .68). *N*_c_ was therefore excluded from further analyses to include the five colonies without *N*_c_ estimates in multivariate models.

Oropharyngeal (saliva) samples were collected by allowing bats to chew on cotton‐tipped wooden swabs (Fisherbrand) for 10 s. Faecal samples were collected using 3‐mm‐diameter rayon‐tipped aluminium swabs (Technical Service Consultants Ltd) dipped in sterile Dulbecco's phosphate buffered saline (Gibco). Swabs were stored in cryovials containing 1 ml RNALater (Ambion) overnight at 4°C before being transferred to dry ice and stored in −70°C freezers, following the manufacturer's instructions.

Two 2‐mm wing biopsy punches were collected and stored in 95% ethanol at 4°C in the field before long‐term storage at −20°C. To examine host population genetic structure and test for associations with viral diversity, host DNA was extracted from wing biopsies and bats were individually genotyped at nine microsatellite loci (Appendix S2; Tables S3–S5).

### 2.3 Environmental variables

Elevation was either recorded in the field (50% of sites) or determined from latitude and longitude coordinates using CGIAR‐SRTM 90‐m resolution data (Farr et al., 2007) obtained using the getdata function from the package raster (Hijmans, 2017) in r version 3.4.2 (R Core Team, 2017). Bioclimatic variables related to temperature and precipitation were gathered from the WorldClim database (Fick & Hijmans, 2017) with a resolution of 5 min of a degree. Climate variables were further analysed by principal component analysis (PCA) to classify sites into three ecoregions: Coast (desert), Andes (mountains) and Amazon (rainforest) (Figure S1). Ecoregion classifications were consistent and modelling results were qualitatively similar when using the full set of 19 bioclimatic variables and when restricting the data set to three variables that captured much of the variation between sites (annual mean temperature [°C], annual precipitation [mm] and annual temperature range [°C]), and therefore only results from the three variable climate data set are presented.

Livestock densities around each site were downloaded from the FAO GLiPHA database for the predominant prey of vampire bats in Peru including cows, pigs, sheep and goats (Bohmann et al., 2018; Food & Agriculture Organization of the United Nations, 2012). The densities for each species were combined and extracted within a 10‐km buffer of each site, as movements between sites have been reported over lesser distances (Trajano, 1996), using the packages maptools (Bivand & Lewin‐Koh, 2017), rgdal (Bivand et al, 2017) and raster.

### 2.4 Metagenomic characterization of viral communities

We generated 48 colony‐level viral communities for vampire bat saliva and faeces using a shotgun metagenomic approach targeting both DNA and RNA viruses (Tables S1 and S6), which has been described in detail previously (Bergner et al., 2019). Nucleic acid was extracted from individual swabs using a Biosprint One for All Vet Kit (Qiagen) and a Kingfisher 96 Flex machine. Extracts from 5 to 10 different individuals per colony were pooled, with 73% of all individuals matched in faecal and saliva pools (Table S1). We generally included samples from 10 individuals in each pool (apart from three faecal and four saliva pools), allowing us to detect viruses present at a minimum of 10% prevalence, on average. The number of individuals included in a pool was not associated with viral richness in faeces (Pearson correlation; all viruses *r* = −.03, *p* = .9; vertebrate‐infecting viruses *r* = −.33, *p* = .1) or saliva (Pearson correlation; all viruses *r* = .14, *p* = .52; vertebrate‐infecting viruses *r* = .14, *p* = .5). Within a colony, individuals were randomly selected for inclusion in metagenomic and host population genetic analyses. We did not evaluate reproducibility of metagenomic results due to the high costs of sequencing, but analyses performed in our previous study showed that viral communities are robustly characterized at the level of sequencing depth typically attained in this study (Bergner et al., 2019). Libraries were combined in equimolar ratios and sequenced in one high output run (version 2, 300 cycles; 150‐bp paired‐end reads) on an Illumina NextSeq500 at the MRC‐University of Glasgow Centre for Virus Research, aiming for 10 million reads per sample.

Sequence data were processed using a custom bioinformatic pipeline (Bergner et al., 2019). Briefly, after read trimming and host mapping, ribosomal reads were removed using ribopicker (Schmieder, Lim, & Edwards, 2012), followed by eukaryotic and bacterial read removal by diamond blastx against a database of RefSeq genomes (Pruitt, Tatusova, & Maglott, 2007). The remaining reads were then de novo assembled with spades (Bankevich et al., 2012) and classified by diamond blastx (Buchfink, Xie, & Huson, 2014) against the GenBank nonredundant (NR) database (Clark, Karsch‐Mizrachi, Lipman, Ostell, & Sayers, 2016), only retaining contigs >300 bp (approximately one read pair length). Contig blastx results were assigned to the lowest taxonomic level using megan Community Edition (Huson et al., 2016) and exported as genus‐level viral communities. Assignments were performed using the lowest common ancestor (LCA) assignment algorithm but altering parameters to include all viral hits passing the filters of the bioinformatic pipeline (maximum e‐value of 0.001).

Viral richness (alpha diversity), equivalent to the number of viral genera, was calculated for each colony using the package vegan (Oksanen, Blanchet, & Friendly, 2017). We estimated both total viral richness and the richness of vertebrate‐infecting viruses using host data from the 2017 ICTV Taxonomy (Adams et al., 2017) as described previously (Bergner et al., 2019). Total viral richness included vertebrate‐infecting viral taxa as well as bacteriophages and plant‐infecting viruses that were unlikely to be actively infecting bats but could nonetheless signal variation in environmental exposures. Differences in viral community composition between colonies (beta diversity) were assessed using Jaccard distances, which were calculated from presence–absence data using vegan. Saliva and faecal viral communities contained largely distinct taxa and were analysed separately. Colony AMA7 was also excluded from analyses as sampling used a different swabbing method which appeared to diminish viral richness.

### 2.5 Variation in viral diversity between ecoregions and effects of geographical and genetic distance

Differences in viral richness between ecoregions were evaluated using Poisson distributed generalized linear models (GLMs). We used the ANOVA function of the car package (Fox & Weisberg, 2011) to calculate the likelihood ratio *χ* ^2^ test (LRT) statistic and assess model significance. All data sets met assumptions of homogeneity of variance. When significant differences were detected by LRT, the multcomp package (Hothorn, Bretz, & Westfall, 2008) was used to perform post‐hoc Tukey pairwise comparisons. Differences in community composition (calculated from presence/absence data) between ecoregions were assessed by permutational multivariate analysis of variance (PERMANOVA) (McArdle & Anderson, 2001) with 10,000 permutations using the adonis function of vegan and visualized by principal coordinate analysis (PCoA) using the pcoa function of the package ape (Paradis, Claude, & Strimmer, 2004).

Effects of geographical and host genetic distance on viral community composition, as measured by Jaccard distances, were evaluated using Mantel tests with 10,000 permutations. Geographical distances between sites were calculated using the function rdist in the package fields (Nychka, Furrer, Paige, & Sain, 2015). Microsatellite genotypes (Appendix S2) were used to calculate pairwise *F*_ST_ values (Nei, 1973) between colonies using the hierfstat package (Goudet & Jombart, 2015).

### 2.6 Local environmental and demographic correlates of viral diversity

Generalized linear models with a Poisson distribution were used to identify demographic and environmental correlates of viral richness (Table 1). For each data set, the global model included environmental and demographic explanatory variables (Proportion Males, Proportion Adults, Other Species Presence, Elevation, Host Genetic Distance, Longitude, Livestock Density and PC1 describing Local Climate Variables) and controlled for sequencing effort (Raw Sequencing Reads). We performed an exploratory analysis finding no effect of human population density (Goldewijk, Beusen, Drecht, & Vos, 2011) on viral richness for either faeces (*R* ^2^ = .001; *p* = .86) or saliva (*R* ^2^ = .04; *p* = .31) and therefore it was excluded from models. Given the small number of observations (*N* = 23 colonies) limiting the statistical power of the full model, 38 submodels per data set were built which restricted the number of explanatory variables per submodel to two and excluded variables with a Pearson correlation coefficient *r* > .5 from the same model (Figure S2). Submodels were compared with Akaike's information criterion corrected for small sample size (AIC_c_). Model‐averaged effect sizes and 95% confidence intervals were calculated for each explanatory variable using the set of GLMs in which the cumulative Akaike weight summed to 0.95 using the dredge and model.avg functions of the package mumin (Bartoń, 2018; Burnham & Anderson, 2002). Effect sizes were standardized using partial standard deviation to account for multicollinearity (Cade, 2015). Relative variable importance was calculated as the sum of Akaike weights across all submodels that included each variable.

Correlations encountered by submodel averaging were confirmed with univariate GLMs examining the effect of each variable individually on viral richness, with *p*‐values corrected for multiple testing using the false discovery rate method with the p.adjust function of the stats package (Benjamini & Hochberg, 1995). For each data set and sample type, a final model was built including only variables for which the model‐averaged effect size significantly differed from 0 to verify consistency in the direction and relative magnitude of effects. No final models exhibited evidence of overdispersion according to the function dispersiontest in the package aer (Kleiber & Zeileis, 2008). As no data sets showed evidence of spatial autocorrelation in raw data or final model residuals according to Moran's i, mixed‐effects models were not used.

Demographic and environmental factors that were significantly correlated with viral richness were also examined for potential effects on viral community composition (presence/absence of viral taxa) using a PERMANOVA with 10,000 permutations and a GLM‐based approach implemented in the mvabund package (Wang, Naumann, Wright, & Warton, 2012). The function manyglm was used to test for differences in viral community composition using a separate logistic regression for each taxon (GLM with binomial error). The function anova.manyglm was then used to test for multivariate significance using the log‐likelihood ratio test statistic and PIT‐trap (probability integral transform residuals) resampling (Warton, Thibaut, & Wang, 2017) with 999 iterations. When multiple variables were tested for the same data set, *p*‐values were corrected using the false discovery rate method as above.

## 3 RESULTS

### 3.1 Taxonomic richness and distribution of viral communities among vampire bat colonies

Metagenomic sequencing of pools from the final 23 sites revealed 108 viral genera from a total of 516,443,646 raw reads (3,534,944–17,852,828 per pool), including 44 vertebrate‐infecting genera as well as other genera that primarily infect plants, invertebrates and bacteria (Table S6; Figure S3). There were 57 viral genera detected across all saliva samples and 77 genera across all faecal samples, with the average vampire bat colony containing 7.9 (range: 1–18) and 10.2 (range: 5–16) viral genera in saliva and faecal samples, respectively (Figure 1b). For each colony, there was a mean of 5.7 vertebrate‐infecting viral genera in saliva (range: 0–12) and 2.3 vertebrate‐infecting viral genera in faeces (range: 0–5). On average, single sites accounted for only 14% of the total viral richness that was discovered in saliva (7.9 out of 57 genera) and only 13% of total viral richness in faeces (10.2 out of 77 genera). Surprisingly, saliva and faecal viral richness within the same site were not correlated (*R* ^2^ = .02; *p* = .51). As expected, we established the presence of some viral genera known to infect vampire bats, including *Lyssavirus*. We also detected full genomes of novel viruses in genera capable of infecting humans such as *Alphacoronavirus* and *Rotavirus*, as well as large contigs of novel *Pircornaviridae* taxa that appeared closely related to *Parechovirus* and *Enterovirus*.

### 3.2 Variation in viral diversity between ecoregions and effects of geographical and genetic distance

**Figure 2:**
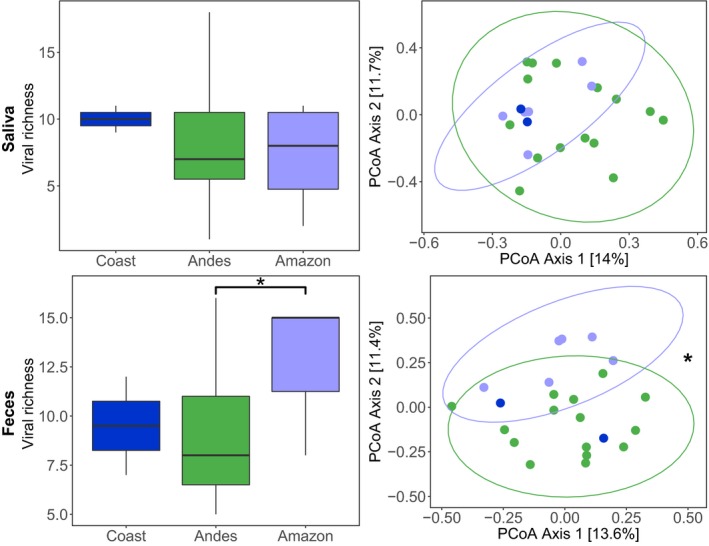
Viral richness and community composition compared across ecoregions. Plots show comparisons across ecoregions in saliva (top panels) and faeces (lower panels). In boxplots, bold lines show the median, and upper and lower hinges show the first and third quartiles. Whiskers extend from the hinge to 1.5 × the interquartile range. An asterisk indicates significance level of post‐hoc Tukey pairwise comparisons (*p < .05). In PCoA plots, circles show the 95% normal probability ellipse for each group and an asterisk indicates communities are significantly different as assessed by PERMANOVA (*p < .05). Colours correspond to different ecoregions within Peru (blue, Coast; green, Andes; purple, Amazon)

For saliva viruses, we rejected the null hypothesis that viral communities are more similar in populations that are more closely connected. Neither total viral richness (Figure 2; LRT; *χ* ^2^ = 1.3; *df* = 2; *p* = .52) nor vertebrate‐infecting viral richness (Figure S4; LRT; *χ* ^2^ = 1.57; *df* = 2; *p* = .46) varied across ecoregions and viral communities clustered neither by ecoregion (Figure 2; Figure S4; Table S7; permanova, all viruses: *F*_2,22_ = 0.91; *p* = .58; vertebrate‐infecting viruses: *F*_2,21_ = 1.18; *p* = .3) nor according to the geographical or genetic distance between bat colonies (Figures S5 and S6). Jaccard distances of saliva community composition often reached the maximum value of 1, implying that communities were locally unique and distance effects were not absent because the same viruses were found everywhere.

In contrast, bats in the Amazon harboured significantly higher faecal viral richness compared to the Andes (Figure 2; LRT; *χ* ^2^ = 6.07; *df* = 2; *p* = .05; Tukey *p* = .03), with a similar trend for vertebrate‐infecting viruses (Figure S4; LRT; *χ* ^2^ = 4.59; *df* = 2; *p* = .1). Viral communities from the Amazon separated from those from the Coast and Andes (Figure 2; Figure S4), but ecoregion explained only 16% of the variation in total viruses (PERMANOVA; *F*_2,22_ = 1.9; *p* = .005) and 14.6% of the variation in vertebrate‐infecting viruses (*F*_2,20_ = 1.5; *p* = .09) (Table S7), suggesting that faecal viral communities were only weakly differentiated based on ecoregion alone. Mantel tests showed that total viral community dissimilarity tended to increase with both the geographical and the genetic distance between bat colonies but highlighted extensive unexplained variation among proximal colonies (Mantel *r* ≤ .25; Figure S5), and no associations with vertebrate‐infecting faecal viruses (Figure S6).

### 3.3 Ecological drivers of viral richness and community composition

**Figure 3:**
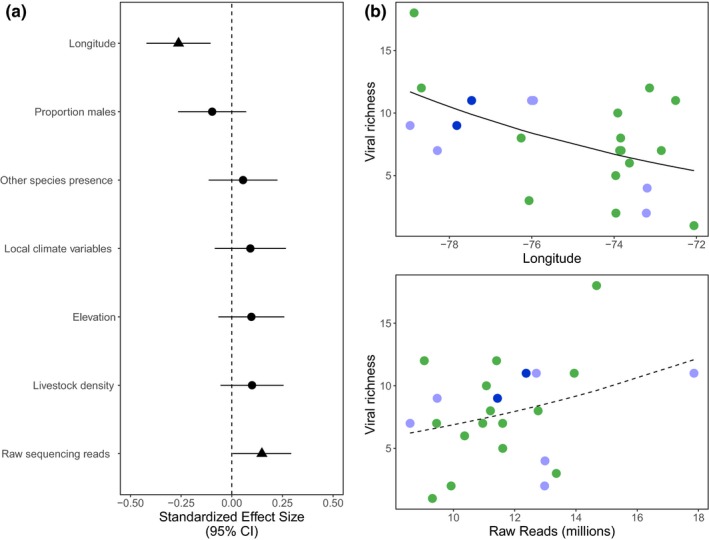
Ecological correlates of viral richness in bat saliva samples. (a) Model‐averaged relationships of demographic and environmental factors with richness and (b) univariate correlations of significant factors. In (a) the model‐averaged effect sizes are shown for each factor across the 95% confidence set of GLMs with 95% confidence intervals. Factors that remained significant in the final model are shown as triangles. The vertical dashed line shows an effect size of zero, such that any confidence intervals overlapping the dashed line indicate a nonsignificant effect of the factor in model‐averaged results. In (b) richness values are plotted for each variable that was significant according to model averaging. Solid lines show GLM predictions for univariate relationships that remained significant following correction for multiple testing, while dashed lines are univariate relationships that were no longer significant after correction. Points are coloured according to ecoregions (blue, Coast; green, Andes; purple, Amazon) [Colour figure can be viewed at http://wileyonlinelibrary.com]

The ecological variables that explained viral richness and community composition differed between faecal and salivary viruses. According to variable selection on submodels, the total richness of saliva viruses was negatively correlated with longitude (relative importance, RI = 1.0) and positively correlated with raw reads (RI = 0.4) (Figure 3; Table S9). Although only longitude was significant in univariate models following *p*‐value correction (Figure 3), both variables were retained in the final model (Table S8; *R* ^2^ = .5). Neither longitude nor raw reads was correlated with total saliva viral community composition (Figure S9; Table S13). For vertebrate‐infecting saliva viruses, longitude was negatively correlated with richness following submodel averaging (RI = 1.0) and remained significant in the univariate richness model (Figure S7; Tables S8 and S10; *R* ^2^ = .34). Longitude was also negatively correlated with the community composition of vertebrate‐infecting saliva viruses (Table S13). Results from both data sets remained consistent even when an outlier colony for high saliva viral diversity (CAJ2) was removed.

**Figure 4:**
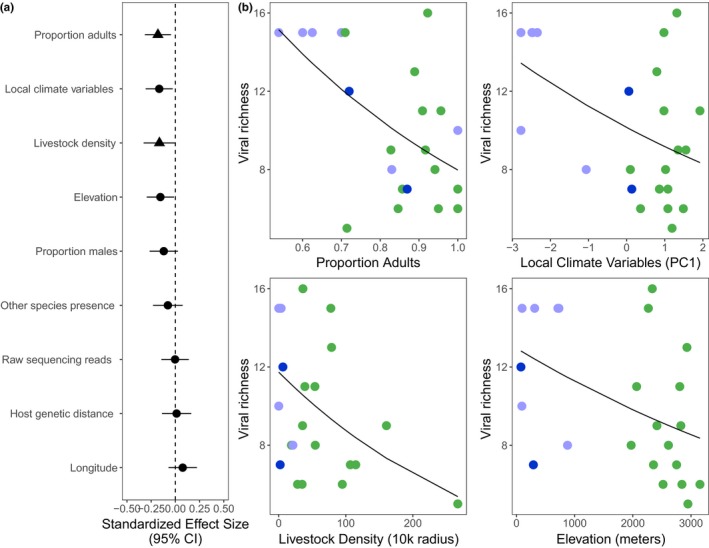
Ecological correlates of viral richness in bat faecal samples. (a) Model‐averaged relationships of demographic and environmental factors with richness and (b) univariate correlations of significant factors. In (a) the model‐averaged effect sizes are shown for each factor across the 95% confidence set of GLMs with 95% confidence intervals. The vertical dashed line shows an effect size of zero, such that any confidence intervals overlapping the dashed line indicate a nonsignificant effect of the factor in model‐averaged results. In (b) richness values are plotted for each variable that was significant according to model averaging. Solid lines show GLM predictions for univariate relationships that remained significant following correction for multiple testing. Points are coloured according to ecoregions (blue, Coast; green, Andes; purple, Amazon) [Colour figure can be viewed at http://wileyonlinelibrary.com]

For faecal viruses, submodels showed that the total viral diversity was negatively correlated with the proportion of adults (RI = 0.62), livestock density (RI = 0.49), elevation (RI = 0.08) and local climate variables (RI = 0.12) (Figure 4; Final model *R* ^2^ = .45; Tables S8–S11). For vertebrate‐infecting viruses, elevation remained negatively correlated with viral richness in submodel averaging (RI = 0.45), but not in the univariate model following *p*‐value correction (Figure S8; Tables S8 and S12; *R* ^2^ = .18). Although no variables were significantly related to community composition when restricting the data set to vertebrate‐infecting viruses, livestock and climate were consistently associated with differences in total faecal virus community composition while the proportion of adults and elevation were variably significant according to the PERMANOVA and GLM analyses (Figure S10; Table S13).

## 4 DISCUSSION

Multispecies comparative analyses and snapshot surveillance of selected viral groups have revealed how interspecific variation in host life history influences viral diversity (Anthony et al., 2015; Luis et al., 2013; Olival et al., 2017; Turmelle & Olival, 2009; Wille et al., 2018). The extent and determinants of variation in viral communities among populations of the same species have remained largely unexplored but have implications for understanding how viral communities will respond to environmental change or human disturbance. Using shotgun metagenomic sequencing of viruses in vampire bat populations across Peru, our study showed extensive variation in viral communities across populations of the same species and rejected a null model of viral similarity being purely driven by spatial proximity of host populations. Instead, an elevational gradient, host age structure and anthropogenic food resources, but not bat colony size, influenced the richness of viral communities in faecal samples. In contrast, the communities of viruses found in saliva were not strongly explained by any tested variables, highlighting major challenges in predicting viral distributions which may depend on virus taxonomy or transmission route.

The richness and community composition of viruses detected in vampire bat saliva was highly variable and largely unpredictable by any spatial, demographic or environmental factor. Indeed, saliva viruses were largely unique to each bat colony, while faecal viral communities were more conserved within ecoregions, with greater similarity among increasingly genetically and geographically proximal bat colonies (Figures S5 and S6). This difference between sample types suggests the possibility that routes of transmission specific to saliva (e.g., biting, licking, grooming) may restrict the extent to which viruses spread across the landscape relative to transmission routes involving faeces (e.g., environmental, faecal–oral). The only factor correlated with saliva virus diversity was longitude (Figure 3; Figure S7; Table S13). In Peru, increasing longitude corresponds to a northwest–southeast gradient, such that sites in the northwest (Cajamarca and Amazonas Departments) had the highest saliva viral diversity. While these sites do not correspond to a single ecoregion, our previous work has highlighted northwest Peru as a corridor for gene flow of vampire bats and rabies virus between the Coast and Andes/Amazon (Streicker et al., 2016) and as a hotspot of mitochondrial haplotype richness (Bohmann et al., 2018). We therefore hypothesize that unusually high mixing of bats from different ecoregions creates a melting pot for saliva viruses. Importantly, northwest Peru is also a hotspot of vampire bat depredation on humans, making regular human exposures to the high diversity of vampire bat salivary viruses encountered here virtually guaranteed (Gilbert et al., 2012; Stoner‐Duncan, Streicker, & Tedeschi, 2014). It is unclear whether the lack of bat‐borne zoonoses reported from this area (apart from rabies virus) reflects the host specificity of most bat viruses, an absence of reporting or insufficient diagnostics for novel human viruses.

We observed a decline in faecal viral richness with increasing elevation (Figure 4) and found that colonies in the low‐elevation Amazon rainforest had higher richness and distinct community composition (Figure 2). Similar elevational gradients have sometimes been observed in bacterial and macrofaunal communities (Fierer et al., 2011; Lomolino, 2001; Wang et al., 2011), but to our knowledge have not been described for viruses. Although the total viral community probably reflects environmental viral taxa as well as those infecting bats, increased viral diversity in the Amazon was also observed in vertebrate‐infecting viruses (Figure S8), suggesting this effect is not only driven by environmental viral diversity. An elevation effect in vampire bats could be explained by the declining diversity of prey, alternative host species or vectors at high elevations, or by factors correlated with elevation that influence the survival of environmentally transmitted viruses. In humans, local climate variables such as temperature and precipitation range, which are correlated with elevation, have been associated with reduced viral richness on a global scale (Guernier et al., 2004). However, that elevation itself was more strongly correlated with vertebrate‐infecting viral richness than environmental variables suggests that other factors that covary with elevation (i.e., community composition of bat species, prey or both) may be more important for viruses actively infecting bats. Indeed, cross‐species transmission is thought to be an important driver of bat viral diversity (Luis et al., 2013, 2015), and bat species richness declines with elevation (Patterson, Pacheco, & Solari, 1996). While our study did not find effects of other bat species on viral richness, it was only possible to measure the presence or absence of other bat species within the same roosts, rather than bat diversity, which might have a stronger effect. Our results also imply that, given expected range shifts towards higher elevations under climate change, we might anticipate novel interactions between hosts, vectors and environmental conditions that could eventually increase diversity in high‐elevation viral communities (Harvell, Altizer, Cattadori, Harrington, & Weil, 2009; Zamora‐Vilchis, Williams, & Johnson, 2012).

We also found that colonies with a higher proportion of juveniles consistently had more diverse faecal viral communities (Figure 4). The importance of juveniles in viral dynamics is well established; births introduce immunologically naïve individuals that facilitate pathogen transmission (Amman et al., 2012; Dietrich et al., 2015; van Dijk et al., 2013; Hayman, 2015). While higher infection rates in juveniles are widely observed at the level of individual pathogens (Anthony et al., 2017; Streicker et al., 2012; Volokhov et al., 2017), to our knowledge, ours is the first report of an age effect on the richness of viral communities. This community‐level effect may be driven by bat demography (e.g., birth pulses or maternal stress) or differential exposure rates among age classes (Cross et al., 2009). For example, independent but subordinate juvenile bats might have elevated exposure to many viruses if they are forced to roost in suboptimal positions within caves (Amman et al., 2012). However, in vampire bats, juveniles remain dependent on their mothers until they are adults (~9–10 months) making this exposure effect unlikely (Greenhall et al., 1983). Consequently, we suggest that the importance of juveniles reflects many pathogens responding similarly to the influx of susceptible individuals, either through heightened transmission following a birth pulse or increased viral shedding by nursing females (Plowright et al., 2008). An effect of susceptible juveniles on viral communities is consistent with observed seasonal variation in individual vampire bat viruses (Streicker et al., 2016) due to birth pulses (Delpietro et al., 2017), and could have implications for viral spillover if shedding of many pathogens simultaneously results in more opportunities for exposure. There are also implications for the use of culling to control vampire bats and their pathogens, as culling is thought to results in a juvenile‐biased age structure (Streicker et al., 2012) and could therefore inadvertently increase viral community richness.

Vampire bats readily exploit livestock as a food source and this has been associated with larger vampire bat populations and colony sizes (Delpietro et al., 1992; Streicker et al., 2012). We therefore predicted that colonies with greater access to livestock would have had higher viral diversity, potentially owing to either density‐dependent transmission or exposure to livestock viruses. Surprisingly, we found the opposite, that vampire bat colonies in areas of high livestock density had lower faecal viral richness (Figure 4). Moreover, colony size was unrelated to viral richness, suggesting that the transmission of many bat viruses may not be density‐dependent (Lloyd‐Smith et al., 2005). If confirmed, this finding would have important implications for managing bat pathogens through culling because artificial reduction of colony sizes would not be expected to reduce prevalence (Blackwood, Streicker, Altizer, & Rohani, 2013). One possible explanation for the observed lower viral diversity could be that the lower diversity of wildlife prey available in areas of high livestock density (Streicker & Allgeier, 2016; Voigt & Kelm, 2006) might reduce vampire bat exposure to a diverse set of viruses. Alternatively, the abundance and availability of livestock prey could enhance bat immunity leading to lower viral diversity, as hypothesized for bacterial infections (Becker et al., 2018).

The weak associations of both faecal and saliva viral communities with spatial or genetic proximity implies that predicting the distributions of viruses or changes to the viral community purely from this null model is unlikely to succeed. As a consequence, snapshots of viral diversity from single locations will not be representative at the host species level and may not even inform whether the virus is present in nearby populations. Adding environmental and demographic variables was able to explain significantly more variance in total saliva and faecal viral richness respectively (50% and 45%, Table S8), although some of the explanatory power for saliva samples was attributable to sequencing effort. This demonstrates that the overall ecological context governing viral exposure plays a key role in shaping viral communities in vampire bats and emphasizes the value of broad‐scale ecological sampling to understand viral community predictability. However, whether the presence of specific viruses can be predicted across populations remains doubtful (Anthony et al., 2015). One possibility would be to include additional variables such as seasonality because the prevalence of some bat viruses varies seasonally (Amman et al., 2012). Here, however, logistical constraints prevented us from sampling most Andean and Amazon sites during the wet season. Furthermore, although our study intended to assess population‐level drivers of viral communities, understanding the individual‐level impacts of factors including age, reproductive status or stress might provide a more complete picture of the determinants of viral diversity.

Although we did not test the ecological and evolutionary consequences of variation in viral diversity in this study, it is probable that some members of the vampire bat viral community identified here have functional importance. For example, viral metagenomics has identified specific viral taxa associated with disease (Duerkop et al., 2018; Hannigan, Duhaime, Ruffin, Koumpouras, & Schloss, 2018; Ly et al., 2014; Norman et al., 2015) and the critical contributions of certain viral groups to overall ecosystem function (Emerson et al., 2018; Sunagawa et al., 2015; Thurber, Payet, Thurber, & Correa, 2017). Follow‐up analyses could include targeting individual viral taxa that are linked with health or disease in other systems, examining associations between variation in viral diversity and host fitness, or testing interactions between viral communities and other components of the host microbiome.

In conclusion, our analysis demonstrates a variety of broad‐scale patterns supporting existing theories from population, behavioural and community ecology, which have been derived from studies of single pathogens or multispecies comparative analyses. Viral communities were distinct across vampire bat populations, suggesting that analyses focusing on single individuals or single populations are unlikely to capture the full extent of species‐level viral diversity and that predicting changes in viral communities is likely to be challenging. Our results illustrate the power of metagenomics as a new approach for finding ecological effects on viral communities at the intraspecific level and demonstrate a combined ecological and metagenomic framework to identify these factors which could be applied in any host–pathogen system.

## AUTHOR CONTRIBUTIONS

L.M.B., C.T., D.J.B. and D.G.S. collected samples; L.M.B., R.J.O., R.B. and D.G.S. conceived the study; L.M.B. performed lab work and wrote the first draft of the manuscript; L.M.B., R.J.O., J.A.B. and D.G.S. analysed data; all authors contributed to the final manuscript.

## Data Availability

Raw sequencing reads have been uploaded to the European Nucleotide Archive (ENA) under accession number PRJEB34487. Scripts used for bioinformatic analyses are available on GitHub (https://github.com/rjorton/Allmond).
