# Supplementary material for: Demographic and environmental drivers of metagenomic viral diversity in vampire bats
Source: Mol Ecol. 2019 Oct 23;29(1):26–39. doi: 10.1111/mec.15250 (PMC7004108; doi:10.1111/mec.15250)
Supplement: Supplementary file 1 [file MEC-29-26-s001.pdf]

Supporting Information: Demographic and environmental drivers of metagenomic viral diversity in vampire bats

Laura M. Bergner[1,2], Richard J. Orton[1,2], Julio A. Benavides[1,3], Daniel J. Becker[4–6], Carlos Tello[7,8], Roman Biek[1], Daniel G. Streicker[1,2]

[1]Institute of Biodiversity, Animal Health and Comparative Medicine, College of Medical, Veterinary and Life Sciences, University of Glasgow, Glasgow, United Kingdom

[2]MRC–University of Glasgow Centre for Virus Research, Glasgow, United Kingdom

[3]Departamento de Ecología, Facultad de Ciencias de la Vida, Universidad Andrés Bello, Santiago, Chile

[4]Odum School of Ecology, University of Georgia, Athens, USA

[5]Center for the Ecology of Infectious Diseases, University of Georgia, Athens, USA

[6]Department of Biology, Indiana University, Bloomington, USA

[7]Association for the Conservation and Development of Natural Resources, Lima, Peru

[8]Yunkawasi, Lima, Peru

Section 1: Population size

Census population size (N_c) was estimated from mark-recapture data for each colony using one of three methods. For sites sampled over multiple years, Cormack-Jolly-Seber models (Cormack 1989) were implemented in the package *Rcapture* (Baillargeon & Rivest 2007). For sites sampled in only one year, the Petersen estimator was calculated for sites with two capture occasions and the Schnabel estimator was calculated for sites with more than two capture occasions, both with Chapman correction (Chapman 1951) in the R package *FSA* (Ogle 2017) (Table S2). Sampling intervals were not consistent between years and across sites, so the same estimator could not be used. Three sites (AMA7, LR2 and LR3) were excluded because roost locations were inaccessible, so sites were sampled around livestock and there were few recaptures. Two other sites (API17 and AYA15) were only sampled using hand nets during the day, making it difficult to account for recapture probability, so colony size was not estimated for those sites. Two datasets were created using the most recent estimates of N_c, as colonies can undergo major changes in N_c over a short number of years (Streicker *et al.* 2012). One dataset included all sites where N_c was estimated and another more conservative dataset included only Petersen or Schnabel estimates from 2016-2017 (Table S2).

The estimate of colony size (N_c) was missing from five sites, so the more and less conservative datasets described above were each analyzed to test for a univariate effect on richness. No relationship was found with either N_c dataset for all combinations of saliva/feces and all viruses/vertebrate-infecting viruses (data not shown) so N_c was excluded from GLMs as an explanatory variable in order to include the five sites missing N_c in models.

Section 2: Host population structure

Nine previously developed microsatellite loci (Piaggio *et al.* 2008) were amplified in two multiplex reactions. For some individuals, loci were amplified according to previously optimized conditions (Piaggio *et al.* 2008), and fragment analysis was performed at the University of Georgia Genomics Facility on an Applied Biosystems 3730xl instrument (Streicker *et al.* 2016). For other individuals, amplification was carried out using a Multiplex PCR Kit (Qiagen) in 15 µL reactions containing a

final concentration of 3 mM MgCl2 and 0.2 µM each primer. PCR conditions were 15 minutes at 95°C, 40 cycles (Panel A) or 35 cycles (Panel B) consisting of 30 seconds at 94°C, 90 seconds at 52°C, and 60 seconds at 72°C, followed by 30 minutes at 60°C. Fragment analysis for these individuals was performed at the University of Dundee DNA Sequencing and Services on an Applied Biosystems 3730xl instrument. Microsatellite scoring was done using either Genemarker v.2.4.0 or the microsatellite plug-in for Geneious v. 7.17 (Kearse *et al.* 2012).

To account for potential scoring discrepancies between labs and microsatellite genotyping errors, 21 individuals were genotyped using both protocols, eight of which were included in the final dataset (the other 13 were from colonies not included in the final dataset). Scores differed in a consistent manner between the two amplification and genotyping methods, as expected given differences between labs and protocols (Ellis *et al.* 2011) and scores from samples genotyped in Glasgow were converted to allow comparison across labs (Table S3). Nineteen individuals were genotyped twice in Glasgow to ensure consistent results across replicates within the same lab. The program PEDANT (Johnson & Haydon 2008) was used to calculate maximum likelihood estimates of genotyping error rate, and to estimate what proportion of errors were due to allelic dropout and false alleles.

The program MicroChecker (Van Oosterhout *et al.* 2004) was used to check for evidence of null alleles within loci or populations. The program FreeNA (Chapuis & Estoup 2007) was also used to calculate null allele frequencies. The inbreeding coefficient F_{IS} (Weir & Cockerham 1984) was estimated for each locus using FSTAT v.2.9.3.2 (Goudet 1995). FSTAT was used to test for significant departures from Hardy-Weinberg equilibrium for within population F_{IS} using 1000 permutations of a randomization test and Bonferroni correction.

Error rates per marker were relatively high for samples re-genotyped across different labs, particularly at loci DeroC12 and DeroD06 (Table S3). In contrast, replicates within Glasgow showed low error rates that were comparable to previous studies (Ellis *et al.* 2011). One locus (DeroH02) showed evidence of null alleles according to both methods, with 13 populations exhibiting evidence of null alleles in MicroChecker. F_{IS} values also deviated significantly from

Hardy-Weinberg equilibrium (HWE) at DeroH02 and DeroD06 (Table S3). Overall there was evidence of genotyping error and null alleles at the three loci DeroC12, DeroD06 and DeroH02. Although the effects of genotyping errors may be less severe in population level analyses compared to analyses reliant on individual identification (Taberlet *et al.* 1999; Pompanon *et al.* 2005), we repeated analyses using a six loci subset, excluding potentially problematic loci to ensure that results were consistent using the two different datasets.

Per locus microsatellite diversity indices were calculated using the program FSTAT, including number of alleles (N_A) and allelic richness (A_R) (Table S4). Observed heterozygosity (H_O) and expected heterozygosity (H_E) were calculated using *adegenet* (Jombart 2008; Jombart & Ahmed 2011) in R and ENA-corrected F_{ST} was calculated using FreeNA. Each pair of loci was tested for evidence of linkage disequilibrium in FSTAT, with no pairs of loci showing evidence of linkage disequilibrium using 1000 permutations of a randomization test and Bonferroni correction.

Per population statistics (Table S5) were calculated using *adegenet* (N_A, A_R, percent missing data per site, H_E, and H_O) and FSTAT (F_{IS}). Two sites (AMA2 and CAJ4) were initially genotyped for larger numbers of individuals than other sites (61 and 88 respectively) but were randomly subsampled to 30 individuals to ensure that unequal sample sizes did not affect other analyses (Puechmaille 2016). Following subsampling, AMA2 deviated significantly from HWE in the 9 loci dataset while no populations deviated significantly in the 6 loci dataset (Table S5).

SUPPLEMENTARY FIGURES

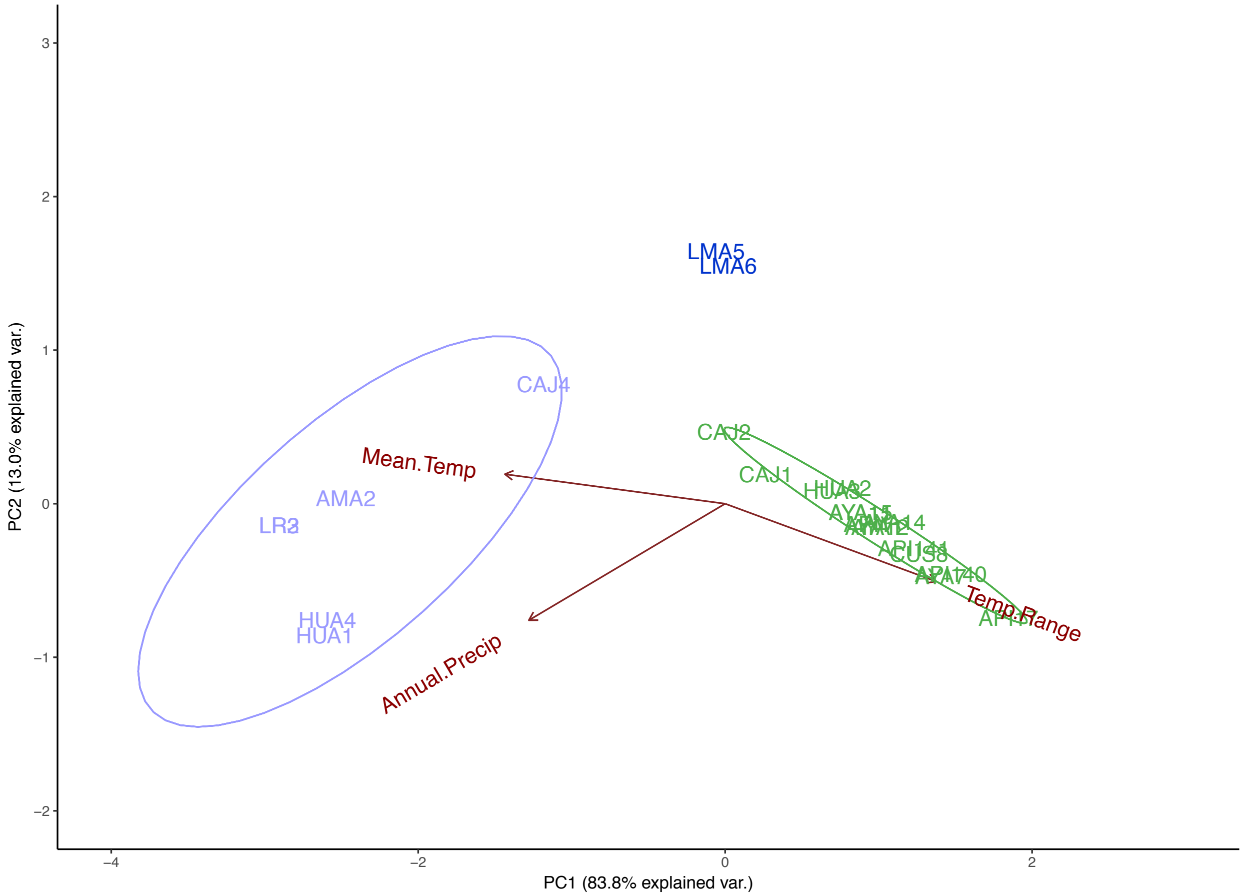

Figure S1. Principal component analysis (PCA) of sites by environmental variables.
The PCA was performed with centering and scaling on the variables annual mean temperature, annual precipitation, and annual temperature range. Sites are colored by ecoregion (blue, Coast; green, Andes; purple, Amazon) and circles show the 95% normal probability ellipse for each group.

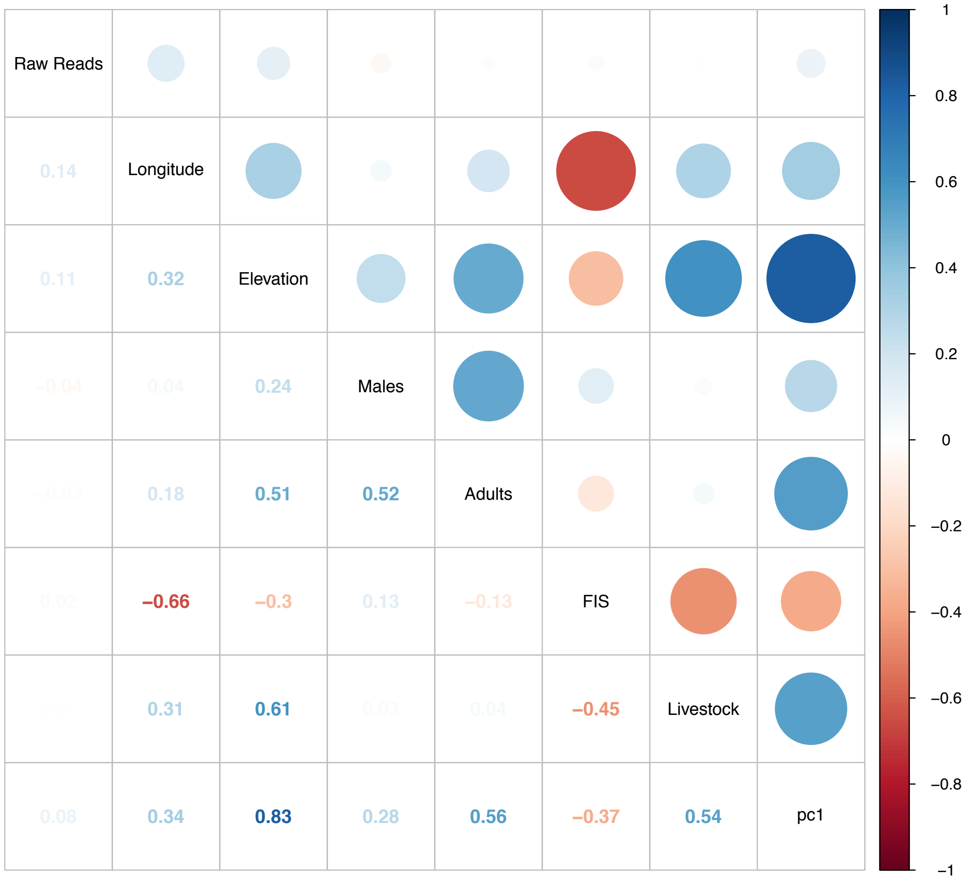

Figure S2. Pearson correlations between variables in viral richness modeling.
Pairwise correlations between all continuous explanatory variables were examined for potential multi-collinearity. Variables with a correlation coefficient of r>0.5 were excluded from the same model in model averaging.

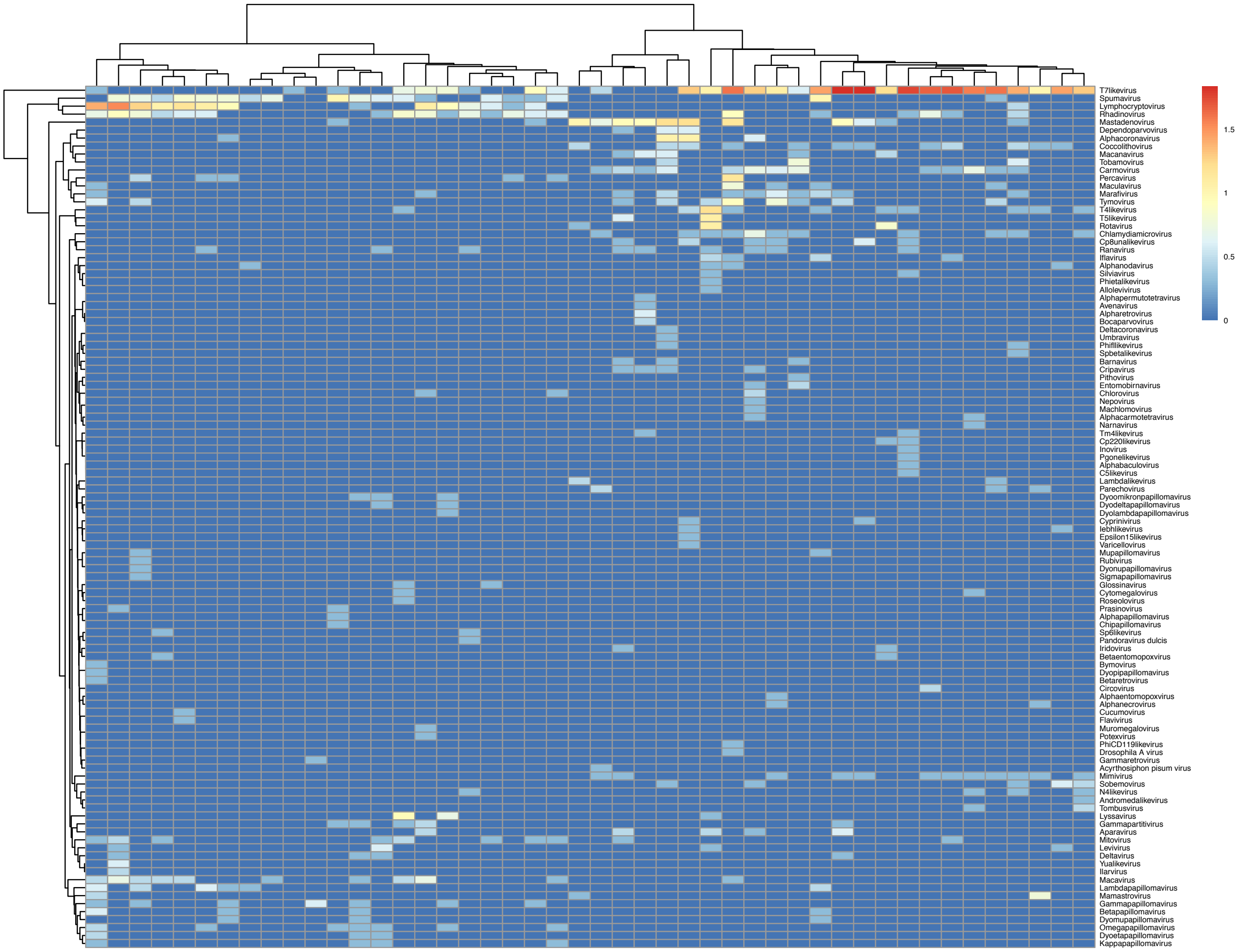

Figure S3. Viral genera detected in vampire bat viral communities

Heatmap of contigs detected per pool where similar rows (viral genera detected together) and columns representing a different colony and sample type (saliva or feces) are clustered according to Ward's method. Number of contigs is depicted on a log10 scale.

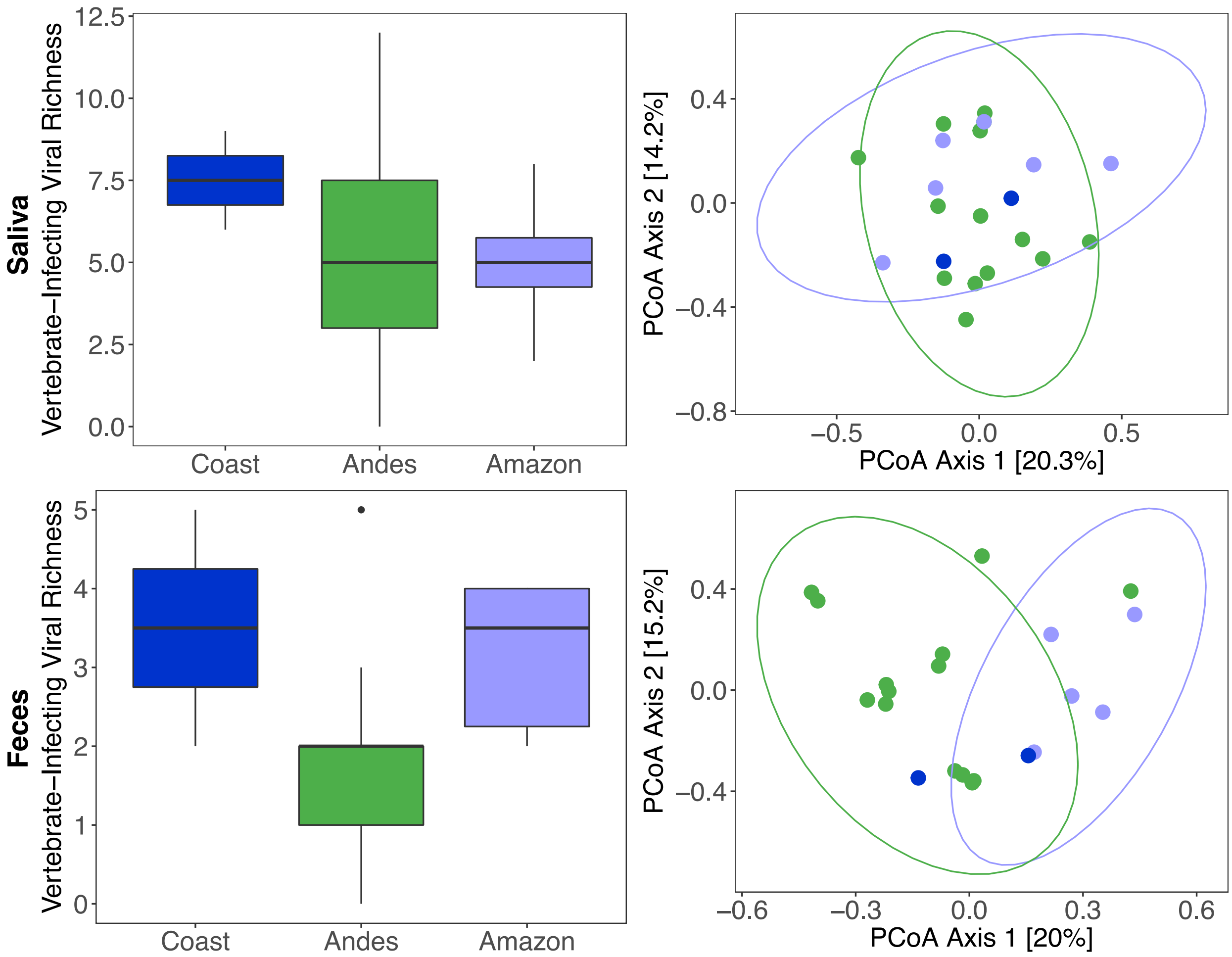

Figure S4. Vertebrate-infecting viral richness and community composition compared across ecoregions. Plots show comparisons of vertebrate-infecting viruses across ecoregions in saliva (top panels) and feces (lower panels). In boxplots, bold lines show the median, and upper and lower hinges show the first and third quartiles. Whiskers extend from the hinge to 1.5 x the inter-quartile range. In PCoA plots, circles show the 95% normal probability ellipse for each group and stars indicate communities are significantly different as assessed by PERMANOVA. Colors correspond to different ecoregions within Peru (blue, Coast; green, Andes; purple, Amazon).

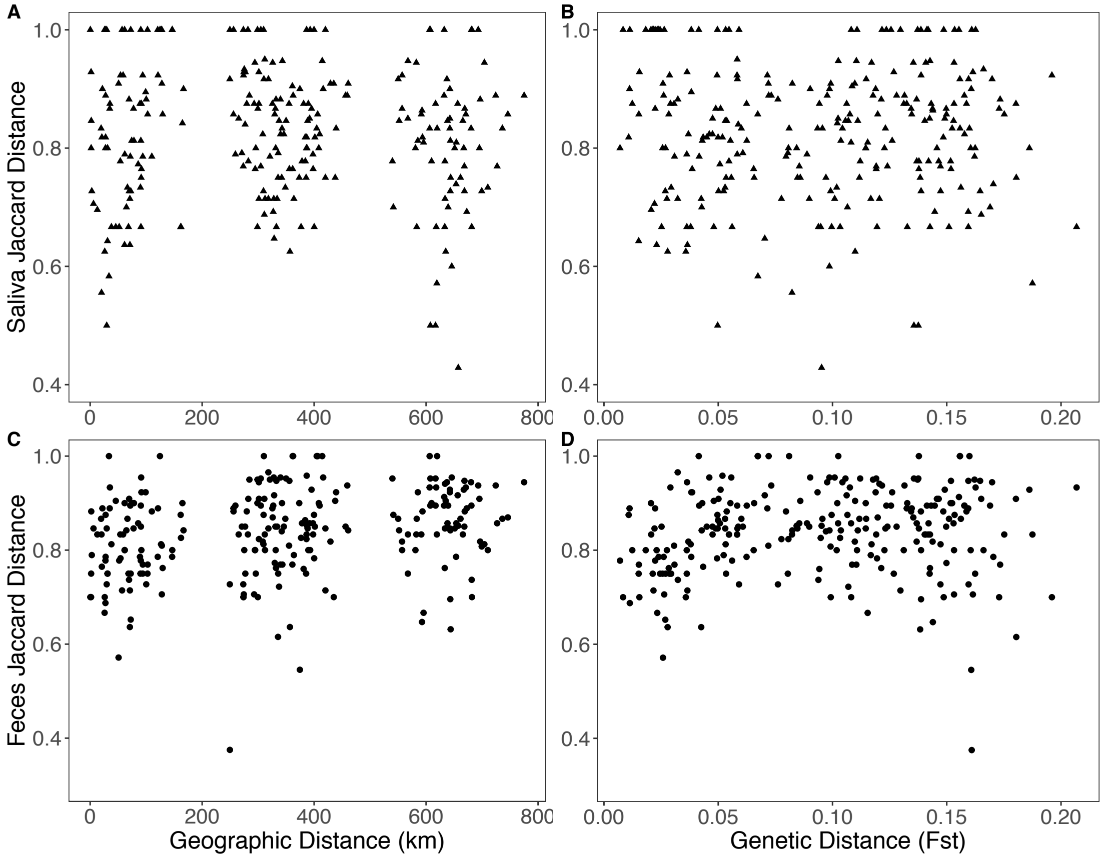

Figure S5. Correlations between viral community distance and geographic or genetic distance. Plots show saliva virus community Jaccard distances compared with colony geographic distance (km) (Panel A; Mantel r=-0.05; p=0.67) and genetic distance F_{ST} calculated using 9 microsatellite loci (Panel B; Mantel r=-0.007; p=0.53), and fecal virus community Jaccard distances correlated with colony geographic distance (km) (Panel C; Mantel r=0.25; p=0.003) and genetic distance F_{ST} calculated using 9 microsatellite loci (Panel D; Mantel r=0.13; p=0.03).

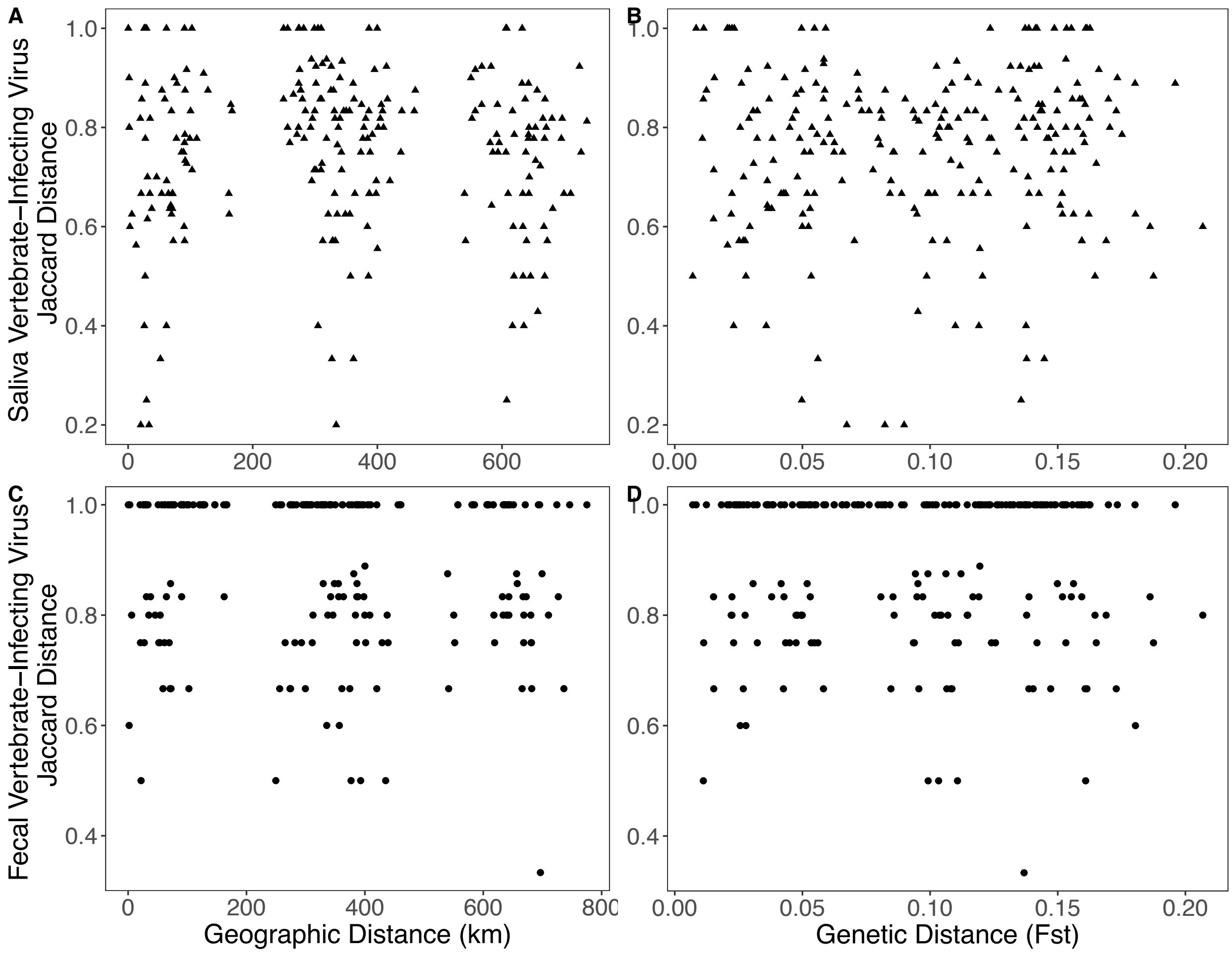

Figure S6. Correlations between vertebrate-infecting viral community distance and geographic or genetic distance. Plots show correlations of vertebrate-infecting saliva virus community Jaccard distances with colony geographic distance (km) (Panel A; Mantel r= 0.006; p= 0.44) and genetic distance F_{ST} calculated using 9 microsatellite loci (Panel B; Mantel r= 0.06; p= 0.19). Fecal vertebrate-infecting virus community Jaccard distances are also compared with colony geographic distance (km) (Panel C; Mantel r= -0.076; p= 0.82) and genetic distance F_{ST} calculated using 9 microsatellite loci (Panel D; Mantel r= -0.015; p= 0.56).

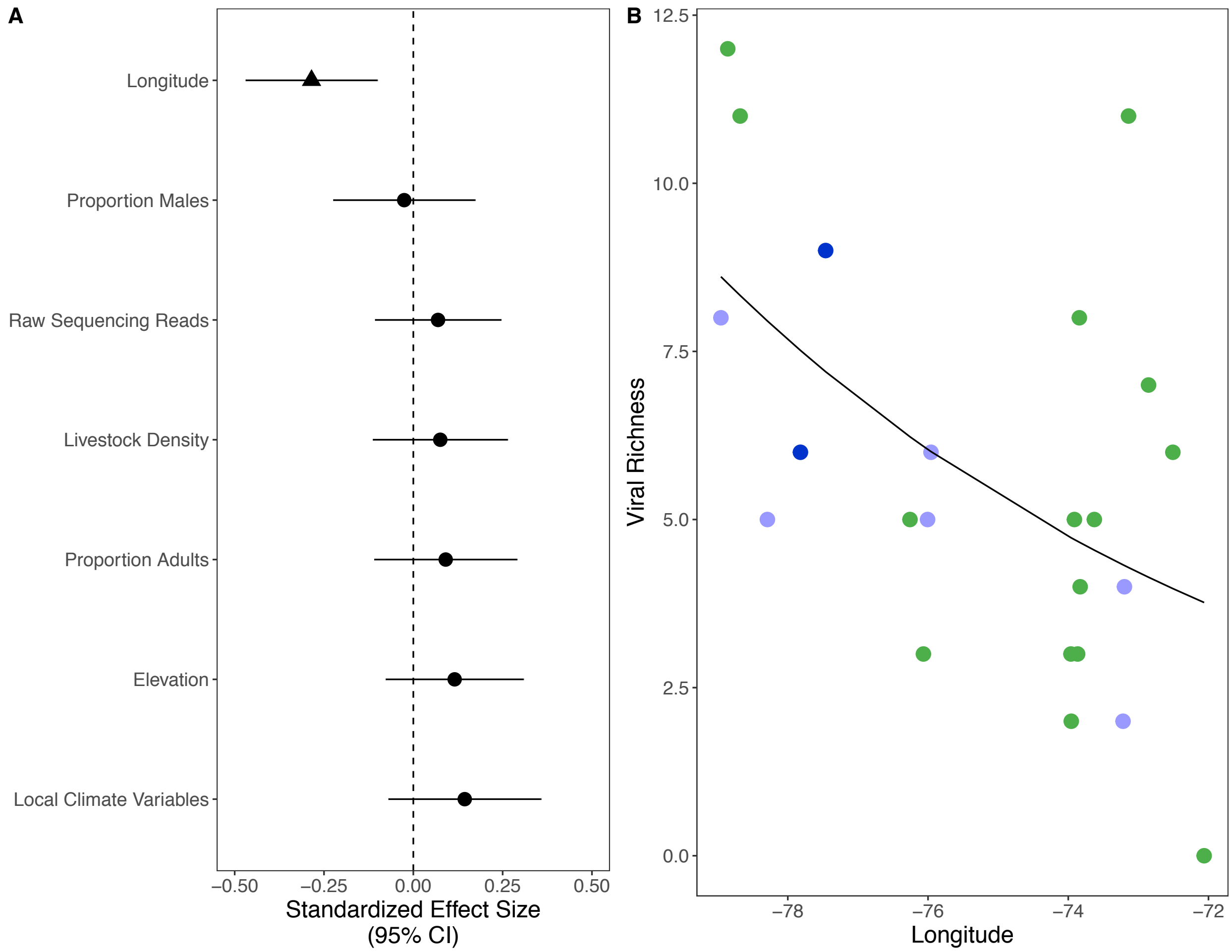

Figure S7. Ecological correlates of vertebrate-infecting viral richness in bat saliva samples. (A) Model averaged relationships of demographic and environmental factors correlated with richness and (B) univariate correlations of significant factors. In panel (A) the model averaged effect sizes are shown for each factor across the 95% confidence set of GLMs with 95% confidence intervals. Factors that remained significant in the final model are shown as triangles. The vertical dashed line shows an effect size of zero, such that any confidence intervals overlapping the dashed line indicate a non-significant effect of the factor in model averaged results. In panel (B) richness values are plotted for each variable that was significant according to model averaging. Solid lines show GLM predictions for univariate relationships that remained significant following correction for multiple testing. Points are colored according to ecoregions (blue, Coast; green, Andes; purple, Amazon).

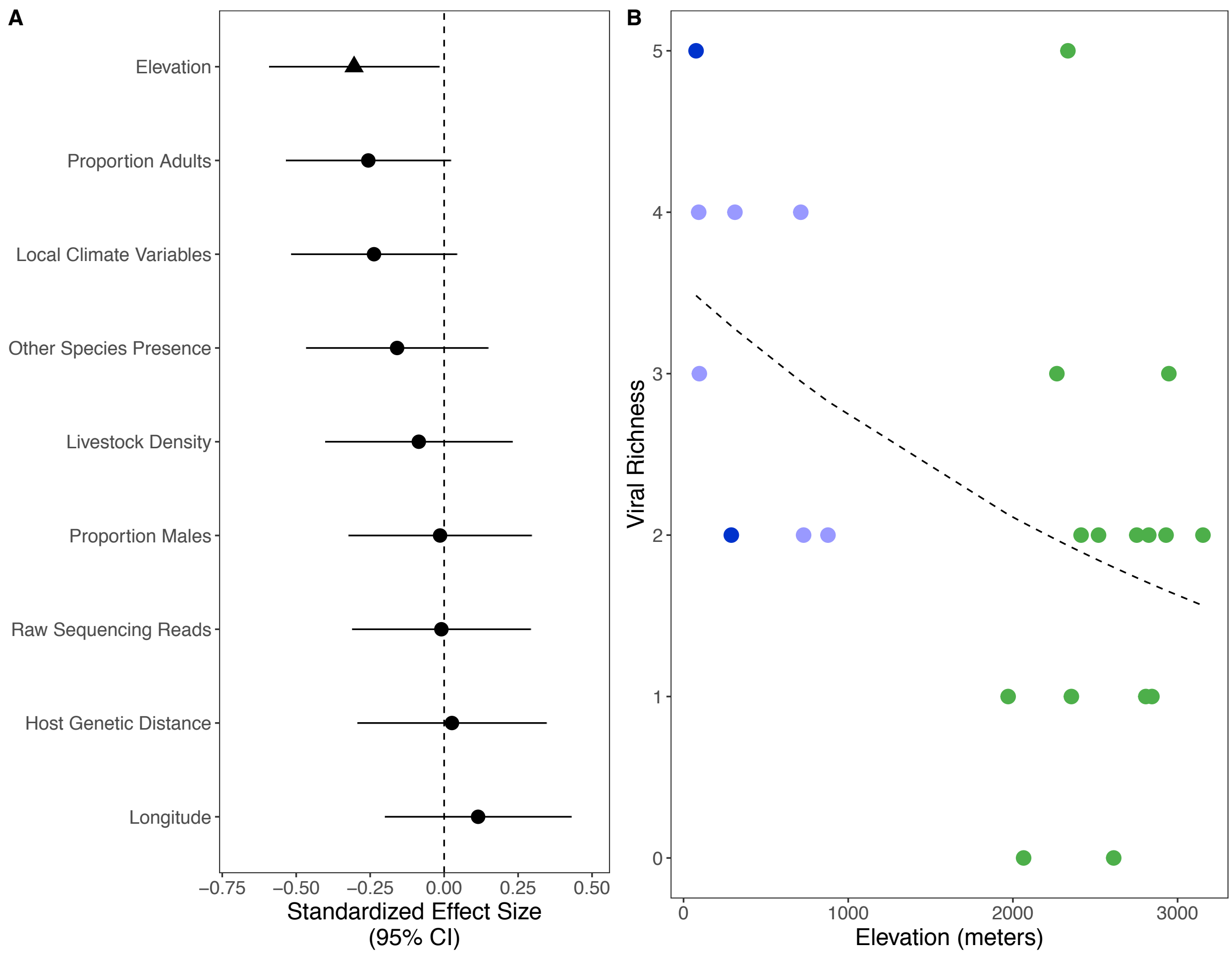

Figure S8. Ecological correlates of vertebrate-infecting viral richness in bat fecal samples. (A) Model averaged relationships of demographic and environmental factors correlated with richness and (B) univariate correlations of significant factors. In panel (A) the model averaged effect sizes are shown for each factor across the 95% confidence set of GLMs with 95% confidence intervals. The vertical dashed line shows an effect size of zero, such that any confidence intervals overlapping the dashed line indicate a non-significant effect of the factor in model averaged results. In panel (B) richness is plotted for each variable that was significant according to model averaging. Dashed lines show univariate relationships that were no longer significant after correction. Points are colored according to ecoregions (blue, Coast; green, Andes; purple, Amazon).

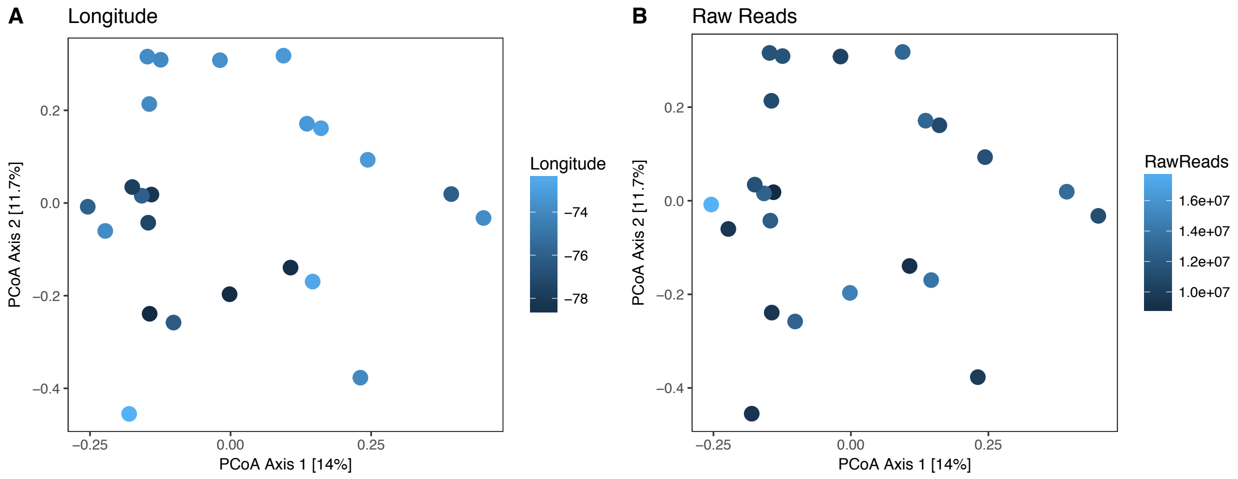

Figure S9. PCoA plots showing the first two axes in which sites have been separated by differences in saliva viral communities and colored according to (A) longitude and (B) number of raw reads.

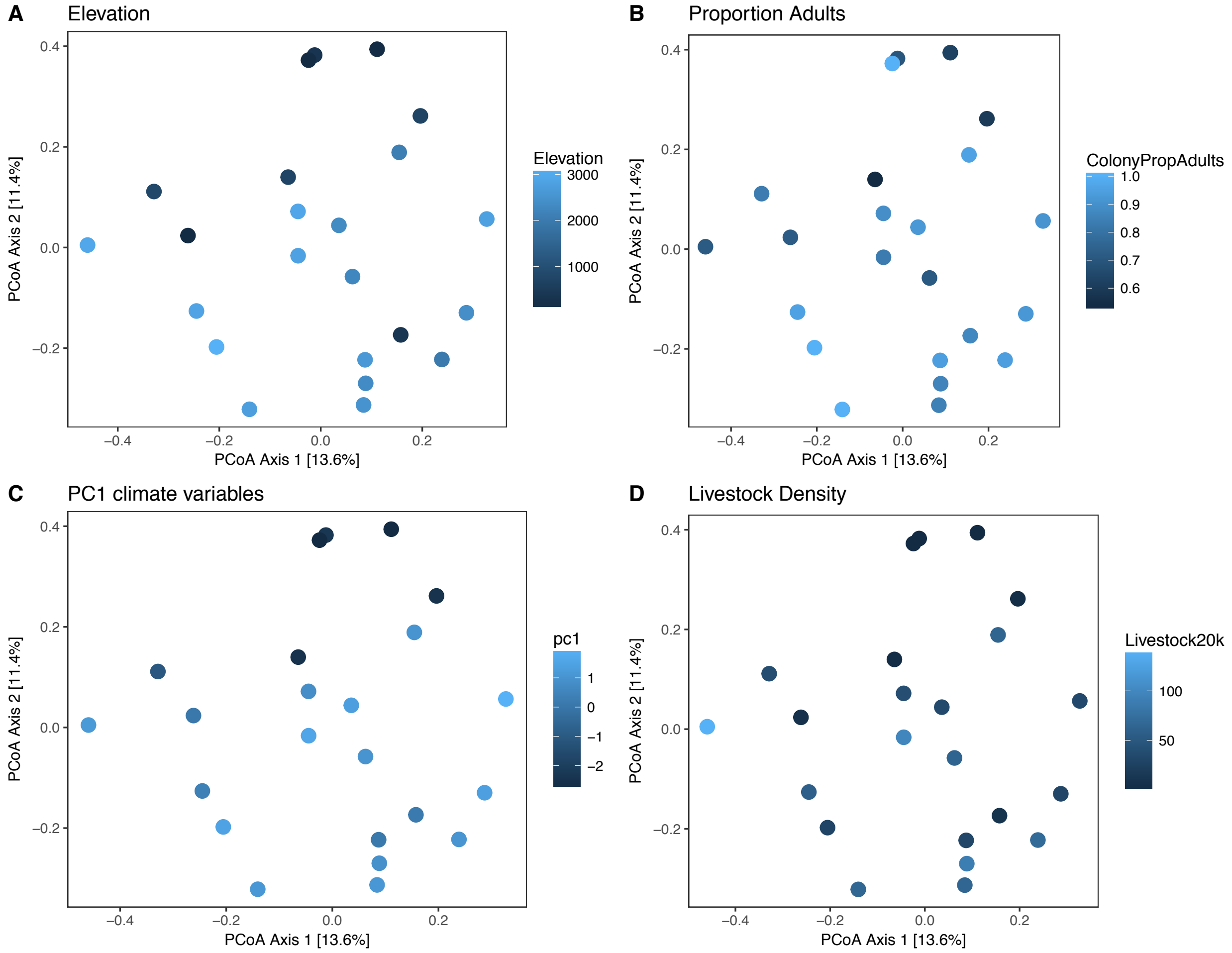

Figure S10. PCoA plots showing the first two axes in which sites have been separated by differences in fecal viral communities and colored according to (A) elevation, (B) proportion of adults (C) local climate variables and (D) livestock density.

SUPPLEMENTARY TABLES

Table S1. Collection details for individual bat samples in metagenomic pools. Bat IDs reflect individuals from which nucleic acid extracts were included in single colony saliva and fecal pools for each site. Month and year of sampling dates are shown for individual bat samples.

Saliva pool	Bat ID†	Month	Year	Fecal pool	Bat ID†	Month	Year
MSV5.1 AMA2	D234	8	2016	MH5.1 AMA2	D203	10	2015
	**D235**	8	2016		D94	10	2015
	**D96**	8	2015		D95	10	2015
	D236	8	2016		**D96**	8	2015
	**D237**	8	2016		D97	10	2015
	8400	12	2016		D98	10	2015
	8401	12	2016		D99	10	2015
	8402	12	2016		SP3	10	2015
	8403	12	2016		**D237**	8	2016
	8405	12	2016		**D235**	8	2016
MSV5.2 AMA7	**D16**	7	2013	MH5.2 AMA7	**D16**	7	2013
	D2	7	2013		D17	7	2013
	D3	7	2013		**D20**	8	2013
	D13	7	2013		D23	8	2013
	D18	7	2013		D25	8	2013
	**D20**	8	2013		**D5**	7	2013
	D24	8	2013		D6	7	2013
	D26	8	2013		D14	8	2013
	**D5**	7	2013		D10	7	2013
	D7	7	2013		D12	7	2013
MSV5.3 API1	**5942**	6	2016	MH5.3 API1	**5942**	6	2016
	**9260**	6	2016		**9260**	6	2016
	**9261**	6	2016		**9261**	6	2016
	**9262**	6	2016		**9262**	6	2016
	**9263**	6	2016		**9263**	6	2016
	9274	6	2016		9266	6	2016
	9275	6	2016		9267	6	2016
	9276	6	2016		9268	6	2016
	9277	6	2016		7603	6	2016
	9278	6	2016		9269	6	2016
MSV5.4 API17	**8325**	7	2016	MH5.4 API17	**8325**	7	2016
	**7959**	7	2016		**7959**	7	2016

	**8326**	7	2016		**8326**	7	2016
	**7986**	7	2016		**7986**	7	2016
	**8327**	7	2016		**8327**	7	2016
	**8328**	7	2016		**8328**	7	2016
	**7980**	7	2016		**7980**	7	2016
	**8329**	7	2016		**8329**	7	2016
	**8330**	7	2016		**8330**	7	2016
	**8331**	7	2016		**8331**	7	2016
MSV5.5 API140	**9237**	5	2016	MH5.5 API140	**9237**	5	2016
	**9238**	5	2016		**9238**	5	2016
	**7132**	5	2016		**7132**	5	2016
	**7141**	5	2016		**7141**	5	2016
	**9239**	5	2016		**9239**	5	2016
	**9240**	5	2016		**9240**	5	2016
	**9241**	5	2016		**9241**	5	2016
	**9242**	5	2016		**9242**	5	2016
	9243	5	2016		9245	5	2016
	**9244**	5	2016		**9244**	5	2016
MSV5.6 API141	**9126**	5	2016	MH5.6 API141	**9126**	5	2016
	**6849**	5	2016		**6849**	5	2016
	**9128**	5	2016		**9128**	5	2016
	**6877**	5	2016		**6877**	5	2016
	**9127**	5	2016		**9127**	5	2016
	**9130**	5	2016		**9130**	5	2016
	**9129**	5	2016		**9129**	5	2016
	**9131**	5	2016		**9131**	5	2016
	**9132**	5	2016		**9132**	5	2016
	**9133**	5	2016		**9133**	5	2016
MSV5.7 AYA1	**7184**	7	2016	MH5.7 AYA1	8228	7	2016
	**8230**	7	2016		7188	7	2016
	**8231**	7	2016		7181	7	2016
	**6933**	7	2016		7802	7	2016
	**6934**	7	2016		8229	7	2016
	8232	7	2016		**7184**	7	2016
	8233	7	2016		**8230**	7	2016
	8234	7	2016		**8231**	7	2016
	8235	7	2016		**6933**	7	2016
	8237	7	2016		**6934**	7	2016
MSV5.8 AYA7	**7064**	7	2016	MH5.8 AYA7	**7064**	7	2016
	**8286**	7	2016		**8286**	7	2016
	**8287**	7	2016		**8287**	7	2016
	**8288**	7	2016		**8288**	7	2016
	**7047**	7	2016		**7047**	7	2016

	8294	7	2016		8289	7	2016
	**8290**	7	2016		**8290**	7	2016
	**8291**	7	2016		**8291**	7	2016
	**8292**	7	2016		**8292**	7	2016
	**8293**	7	2016		**8293**	7	2016
MSV5.9 AYA11	**8201**	7	2016	MH5.9 AYA11	**8201**	7	2016
	**8202**	7	2016		**8202**	7	2016
	**8203**	7	2016		**8203**	7	2016
	**7082**	7	2016		**7082**	7	2016
	**8204**	7	2016		**8204**	7	2016
	8217	7	2016		7864	7	2016
	8218	7	2016		**8205**	7	2016
	**8206**	7	2016		**8206**	7	2016
	**8205**	7	2016		8207	7	2016
	8221	7	2016		8208	7	2016
MSV5.10 AYA12	**8222**	7	2016	MH5.10 AYA12	**8222**	7	2016
	**7169**	7	2016		**7169**	7	2016
	**8223**	7	2016		**8223**	7	2016
	**8224**	7	2016		**8224**	7	2016
	**8225**	7	2016		**8225**	7	2016
	**8226**	7	2016		**8226**	7	2016
	**8227**	7	2016		**8227**	7	2016
MSV5.11 AYA14	**8297**	7	2016	MH5.11 AYA14	**8297**	7	2016
	**8298**	7	2016		**8298**	7	2016
	**8299**	7	2016		**8299**	7	2016
	**8300**	7	2016		**8300**	7	2016
	**8301**	7	2016		**8301**	7	2016
	8320	7	2016		8302	7	2016
	8321	7	2016		8303	7	2016
	8322	7	2016		8304	7	2016
	8323	7	2016		8305	7	2016
	7774	7	2016		8306	7	2016
MSV5.12 AYA15	**9286**	6	2016	MH5.12 AYA15	**9286**	6	2016
	**9284**	6	2016		**9284**	6	2016
	**9285**	6	2016		**9285**	6	2016
	**9287**	6	2016		**9287**	6	2016
	**9288**	6	2016		**9288**	6	2016
	**9289**	6	2016		**9289**	6	2016
	**9290**	6	2016		**9290**	6	2016
	**9291**	6	2016		**9291**	6	2016
	**9292**	6	2016		**9292**	6	2016
	**9293**	6	2016		**9293**	6	2016
MSV5.13	**8080**	11	2016	MH5.13	**8080**	11	2016

CAJ1	**8081**	11	2016	CAJ1	**8081**	11	2016
	**8082**	11	2016		**8082**	11	2016
	**8083**	11	2016		**8083**	11	2016
	**4156**	11	2016		**4156**	11	2016
	**8084**	11	2016		**8084**	11	2016
	**8085**	11	2016		**8085**	11	2016
	**8086**	11	2016		**8086**	11	2016
	**8087**	11	2016		**8087**	11	2016
	**8088**	11	2016		**8088**	11	2016
MSV5.14 CAJ2	**8096**	12	2016	MH5.14 CAJ2	**8096**	12	2016
	**8097**	12	2016		**8097**	12	2016
	**8098**	12	2016		**8098**	12	2016
	**8099**	12	2016		**8099**	12	2016
	**8100**	12	2016		**8100**	12	2016
	8109	12	2016		8101	12	2016
	8110	12	2016		8102	12	2016
	8111	12	2016		8103	12	2016
	8112	12	2016		8104	12	2016
	4693	12	2016		6181	12	2016
MSV5.15 CAJ4	8361	12	2016	MH5.15 CAJ4	D83	10	2015
	8362	12	2016		D84	10	2015
	8363	12	2016		D85	10	2015
	8364	12	2016		D86	10	2015
	8365	12	2016		D87	10	2015
	8366	12	2016		D88	10	2015
	8367	12	2016		D89	10	2015
	8368	12	2016		D90	10	2015
	8369	12	2016		D91	10	2015
	8370	12	2016		D92	10	2015
MSV5.16 CUS8	**9216**	5	2016	MH5.16 CUS8	**9216**	5	2016
	**9217**	5	2016		**9217**	5	2016
	**7503**	5	2016		**7503**	5	2016
	**9218**	5	2016		**9218**	5	2016
	**9219**	5	2016		**9219**	5	2016
	**7995**	5	2016		**7995**	5	2016
	**9220**	5	2016		**9220**	5	2016
	**9221**	5	2016		**9221**	5	2016
	**9222**	5	2016		**9222**	5	2016
	**9223**	5	2016		**9223**	5	2016
MSV5.17 HUA1	**8009**	10	2016	MH5.17 HUA1	**8009**	10	2016
	**8010**	10	2016		**8010**	10	2016
	**8011**	10	2016		**8011**	10	2016
	**8012**	10	2016		**8012**	10	2016

	**8013**	10	2016		**8013**	10	2016
	**8014**	10	2016		**8014**	10	2016
	**8015**	10	2016		**8015**	10	2016
	**8016**	10	2016		**8016**	10	2016
	**8017**	10	2016		**8017**	10	2016
	**8018**	10	2016		**8018**	10	2016
MSV5.18 HUA2	**8332**	10	2016	MH5.18 HUA2	**8332**	10	2016
	**8333**	10	2016		**8333**	10	2016
	**6750**	10	2016		**6750**	10	2016
	**8334**	10	2016		**8334**	10	2016
	**8335**	10	2016		**8335**	10	2016
	**7707**	10	2016		**7707**	10	2016
	**8336**	10	2016		**8336**	10	2016
	**8337**	10	2016		**8337**	10	2016
	**8338**	10	2016		**8338**	10	2016
	**8339**	10	2016		**8339**	10	2016
MSV5.19 HUA3	**8003**	10	2016	MH5.19 HUA3	**8003**	10	2016
	**8004**	10	2016		**8004**	10	2016
	**8005**	10	2016		**8005**	10	2016
	**8006**	10	2016		**8006**	10	2016
	**8007**	10	2016		**8007**	10	2016
	**7738**	10	2016		**7738**	10	2016
	**9050**	10	2016		**9050**	10	2016
	**9022**	10	2016		**9022**	10	2016
	**8008**	10	2016		**8008**	10	2016
MSV5.20 HUA4	**8022**	10	2016	MH5.20 HUA4	**8022**	10	2016
	**8023**	10	2016		**8023**	10	2016
	**8024**	10	2016		**8024**	10	2016
	**8025**	10	2016		**8025**	10	2016
	**8026**	10	2016		**8026**	10	2016
	**8027**	10	2016		**8027**	10	2016
	**8028**	10	2016		**8028**	10	2016
	**8029**	10	2016		**8029**	10	2016
	**8030**	10	2016		**8030**	10	2016
	**8031**	10	2016		**8031**	10	2016
MSV5.21 LMA5	**9110**	4	2016	MH5.21 LMA5	**9110**	4	2016
	**7681**	4	2016		**7681**	4	2016
	**3859**	4	2016		**3859**	4	2016
	**9111**	4	2016		**9111**	4	2016
	**9112**	4	2016		**9112**	4	2016
MSV5.22 LMA6	**9083**	4	2016	MH5.22 LMA6	**9083**	4	2016
	**2926**	4	2016		**2926**	4	2016
	**4557**	4	2016		**4557**	4	2016

	**9084**	4	2016		**9084**	4	2016
	**9085**	4	2016		**9085**	4	2016
	8072	11	2016		8060	11	2016
	5367	11	2016		8061	11	2016
	2941	11	2016		8062	11	2016
	8074	11	2016		8063	11	2016
	8075	11	2016		8064	11	2016
MSV5.23 LR2	**D256**	9	2016	MH5.23 LR2	D39	9	2015
	D257	9	2016		D57	9	2015
	D258	9	2016		D74	9	2015
	D259	9	2016		D75	9	2015
	**D260**	9	2016		D76	9	2015
	**D261**	9	2016		D77	9	2015
	D262	9	2016		**D59**	5	2016
	**D0059**	5	2016		**D256**	9	2016
					**D260**	9	2016
					**D261**	9	2016
MSV5.24 LR3	**D249**	9	2016	MH5.24 LR3	**D242**	9	2016
	**D248**	9	2016		**D244**	9	2016
	**D243**	9	2016		**D243**	9	2016
	**D246**	9	2016		**D246**	9	2016
	**D245**	9	2016		**D248**	9	2016
	**D244**	9	2016		**D247**	9	2016
	**D247**	9	2016		**D249**	9	2016
	**D250**	9	2016		**D245**	9	2016
	**D242**	9	2016		**D250**	9	2016
	D216	5	2015		D73	9	2015

†Bat IDs in bold show individual IDs for those that were included in pools for both fecal and saliva swabs.

Table S2. Colony size (*Nc*) estimates and other species presence at vampire bat colonies. Colony size estimates were generated based on mark-recapture data using different methods depending on the data. The *Nc* estimate from the most recent year is presented along with the year of that estimate. The recorded presence of other bat species within each colony and the estimated count of other species is also presented.

Site†	N_c method	N_c estimate	N_c estimate year	Other species recorded (estimated species count)
AMA7	NA	NA	NA	N (0)
AMA2	Petersen	11	2012	Y (5)
**API1**	Petersen	74	2016	Y (2)
API17	NA	NA	NA	N (0)
API140	CJS	536	2015	N (0)
**API141**	Petersen	322	2016	N (0)
**AYA1**	Schnabel	276	2016	N (0)
**AYA7**	Petersen	25	2016	Y (2)
**AYA11**	Petersen	39	2016	Y (1)
AYA12	CJS	24	2015	Y (2)
**AYA14**	Petersen	94	2017	Y (1)
AYA15	NA	NA	NA	Y (1)
**CAJ1**	Petersen	22	2016	Y (2)
**CAJ2**	Petersen	77	2016	Y (3)
**CAJ4**	Petersen	312	2016	Y (6)
**CUS8**	Schnabel	168	2017	N (0)
HUA1	CJS	122	2015	Y (5)
HUA2	CJS	47	2013	N (0)
HUA3	Petersen	288	2014	N (0)
HUA4	CJS	31	2013	Y (4)
LMA5	CJS	510	2015	N (0)
**LMA6**	Schnabel	207	2016	Y (1)
LR2	NA	NA	NA	Y (6)
LR3	NA	NA	NA	Y (4)

†Sites in bold are in the more conservative dataset including only Petersen or Schnabel estimates from 2016-2017

Table S3. Microsatellite error rate estimates, null alleles and F_{IS} estimates per locus. Conversion shows the number of base pairs different between microsatellite loci scored in different labs. Error rate estimates across and between labs were calculated using PEDANT. Number of populations showing evidence of null alleles were based on MicroChecker. Average null allele frequency across populations were based on FreeNA.

		Across labs		Within lab				
Locus	Convert	Allelic dropout	False alleles	Allelic dropout	False alleles	Null pops	Null freq	F_{IS}†
DeroB03	-1	0	0	0.000001	0	1	0.028	0.038
DeroB10	--	0	0.05	0	0	2	0.024	0.046
DeroB11	--	--	--	--	--	0	0.001	-0.048
DeroC12	2	0.26	0.24	0.000002	0	0	0.014	-0.026
DeroD06	2	0.7	0.05	--	--	4	0.034	**0.405**
DeroC07	2	0	0	0.000001	0	1	0.022	0.023
DeroD12	1	0	0	0	0	1	0.013	-0.01
DeroG12	1	0	0	0.000001	0.000001	3	0.036	0.056
Dero H02	2	0	0	0	0	13	0.105	**0.509**

†Significant departure from Hardy-Weinberg equilibrium indicated in bold; p-values were adjusted using the Bonferroni correction (adjusted α=0.006)

Table S4. Microsatellite per locus diversity indices for vampire bats. Diversity indices were calculated using the programs FSTAT and FreeNA, and the R package adegenet. Per locus data are shown for all individuals combined across colonies.

Locus	Number of alleles (N_A)	Allelic richness (A_R)	Expected heterozygosity (H_E)	Observed heterozygosity (H_O)	F_{ST} ENA†
DeroB03	7	3.37	0.61	0.47	0.19
DeroB10	14	6.82	0.88	0.76	0.1
DeroB11	3	1.09	0.01	0.01	0.04
DeroC12	14	5.8	0.83	0.76	0.11
DeroD06	6	1.51	0.1	0.05	0.20
DeroC07	12	4.53	0.72	0.51	0.28
DeroD12	15	4.06	0.59	0.53	0.11
DeroG12	18	5.05	0.72	0.61	0.11
Dero H02	13	3.32	0.45	0.18	0.3

†F_{ST} corrected based on the ENA method to correct for the presence of null alleles (Chapuis & Estoup 2007)

Table S5. Microsatellite per population statistics for vampire bat colonies. Diversity indices were calculated using the program FSTAT and the R package adegenet. Data are shown for each colony separately.

Site	N	Number of alleles (N_A)	Allelic richness (A_R)	Percent missing d ata	Expected heterozygosity (H_E)	Observed heterozygosity (H_O)	F_{IS}†
AMA7	25	53	38.50	0	0.60	0.59	0.038
AMA2	30	56	40.82	6.3	0.63	0.56	**0.134**
API1	29	35	26.70	7.66	0.43	0.47	-0.067
API140	29	32	28.15	6.51	0.44	0.45	0.017
API141	29	41	27.82	0.38	0.44	0.43	0.015
API17	34	37	24.75	0	0.38	0.38	0.026
AYA1	29	30	23.31	0	0.33	0.35	-0.059
AYA11	29	26	23.55	0.38	0.34	0.38	0.096
AYA12	30	31	24.12	0	0.34	0.33	-0.083
AYA14	30	31	22.00	1.48	0.31	0.34	0.037
AYA15	29	29	24.05	0	0.34	0.35	-0.091
AYA7	24	29	20.86	0.46	0.26	0.24	0.006
CAJ1	31	48	34.32	7.89	0.53	0.48	0.117
CAJ2	13	39	31.49	6.84	0.48	0.43	0.156
CAJ4	30	48	35.40	3.33	0.54	0.49	0.103
CUS8	25	33	23.64	0	0.32	0.32	0.02
HUA1	24	50	37.42	4.17	0.58	0.53	0.124
HUA2	21	38	29.70	1.59	0.49	0.39	0.222
HUA3	22	36	30.24	8.59	0.49	0.44	0.125
HUA4	21	45	36.16	2.65	0.58	0.55	0.077
LMA5	28	38	27.99	1.19	0.48	0.41	0.164
LMA6	29	36	27.41	2.68	0.50	0.46	0.084
LR2	15	45	39.10	0	0.60	0.59	0.058
LR3	18	52	41.60	0	0.61	0.58	0.072

†Significant departure from Hardy-Weinberg equilibrium indicated in bold; p-values were adjusted using the Bonferroni correction (adjusted α= 0.00023)

Table S6. Single colony pools sequenced to characterize viral communities in vampire bats. Pools were created by combining nucleic acid from up to 10 individual swab samples from the same sample type and the same colony.

Pool ID†	Sample Type	Colony‡	Raw Reads	Viral Reads	Proportion Viral
AMA7_F	Feces	AMA7	13,458,777	54	0.000004
AMA7_SV	Saliva	AMA7	10,908,218	160	0.000015
AMA2_F	Feces	AMA2	10,867,145	16,002	0.001473
AMA2_SV	Saliva	AMA2	8,583,589	360	0.000042
API1_F	Feces	API1	11,050,071	1,286	0.000116
API1_SV	Saliva	API1	11,605,602	210	0.000018
API17_F	Feces	API17	9,323,302	8,084	0.000867
API17_SV	Saliva	API17	9,312,286	128	0.000014
API140_F	Feces	API140	8,455,451	17,522	0.002072
API140_SV	Saliva	API140	11,407,649	722	0.000063
API141_F	Feces	API141	13,278,728	155,696	0.011725
API141_SV	Saliva	API141	10,955,775	558	0.000051
AYA1_F	Feces	AYA1	7,098,210	30,434	0.004288
AYA1_SV	Saliva	AYA1	9,922,871	94	0.000009
AYA7_F	Feces	AYA7	11,345,890	13,006	0.001146
AYA7_SV	Saliva	AYA7	10,363,555	490	0.000047
AYA11_F	Feces	AYA11	8,590,173	6,680	0.000778
AYA11_SV	Saliva	AYA11	11,207,240	324	0.000029
AYA12_F	Feces	AYA12	13,458,223	51,540	0.003830
AYA12_SV	Saliva	AYA12	11,608,698	110	0.000009
AYA14_F	Feces	AYA14	9,592,865	2,024	0.000211
AYA14_SV	Saliva	AYA14	11,075,284	1,178	0.000106
AYA15_F	Feces	AYA15	7,492,336	35,392	0.004724
AYA15_SV	Saliva	AYA15	9,448,671	254	0.000027
CAJ1_F	Feces	CAJ1	8,187,011	1,712	0.000209
CAJ1_SV	Saliva	CAJ1	9,047,393	262	0.000029
CAJ2_F	Feces	CAJ2	8,829,558	9,534	0.001080
CAJ2_SV	Saliva	CAJ2	14,671,986	1,122	0.000076
CAJ4_F	Feces	CAJ4	8,532,268	3,608	0.000423
CAJ4_SV	Saliva	CAJ4	9,468,189	316	0.000033
CUS8_F	Feces	CUS8	14,834,175	19,192	0.001294
CUS8_SV	Saliva	CUS8	13,942,320	990	0.000071
HUA1_F	Feces	HUA1	9,362,178	30,676	0.003277
HUA1_SV	Saliva	HUA1	17,852,828	606	0.000034
HUA2_F	Feces	HUA2	13,876,749	6,988	0.000504
HUA2_SV	Saliva	HUA2	12,764,201	416	0.000033
HUA3_F	Feces	HUA3	3,534,944	19,188	0.005428
HUA3_SV	Saliva	HUA3	13,357,838	210	0.000016
HUA4_F	Feces	HUA4	16,396,134	17,064	0.001041

HUA4_SV	Saliva	HUA4	12,705,637	448	0.000035
LMA5_F	Feces	LMA5	8,103,620	6,390	0.000789
LMA5_SV	Saliva	LMA5	11,437,853	234	0.000020
LMA6_F	Feces	LMA6	7,712,739	392	0.000051
LMA6_SV	Saliva	LMA6	12,372,323	168	0.000014
LR2_F	Feces	LR2	6,963,077	2,614	0.000375
LR2_SV	Saliva	LR2	12,988,290	210	0.000016
LR3_F	Feces	LR3	6,115,567	1,164	0.000190
LR3_SV	Saliva	LR3	12,976,159	96	0.000007

†Pool IDs reflect the colony and sample type (F, feces; SV, saliva)
‡Colony names reflect the department (AMA, Amazonas; API, Apurímac; AYA, Ayacucho; CAJ, Cajamarca; CUS, Cusco; HUA, Huánuco; LMA, Lima; LR, Loreto). Locations are shown in Figure 1.

Table S7. Multivariate PERMANOVA testing the effect of ecoregion on viral community composition. Separate tests were performed for each sample type (saliva and feces) and virus data set (all viruses and vertebrate-infecting).

	d.f.	*F*	R^2	*P*-value†
All Virus Community Saliva	2,22	0.91	0.08	0.58
Vertebrate-infecting Virus Community Saliva	2,21	1.18	0.11	0.3
All Virus Community Feces	2,22	1.93	0.16	**0.005**
Vertebrate-infecting Virus Community Feces	2,20	0.15	0.15	0.09

†Significant p-values shown in bold

Table S8. Viral richness final models with variables significant in model averaging. All variables included in final models had a model-averaged effect size that significantly differed from 0. Final model results were examined to verify that direction and relative magnitude of effect sizes were consistent with those from model averaging and univariate models. Final models were constructed for each sample type (saliva and feces) and virus dataset (all viruses and vertebrate-infecting).

	Best model	R^2	Adj R^2	Variable	Estimate (Std error)	z	P-value	Partial R^2
Saliva All Richness	Richness~ Longitude + RawReads	0.5	0.5	Longitude	-0.11 (0.03)	-3.45	**0.001**	0.4
				Raw Reads	0.00000007 (0.00000003)	2.14	**0.03**	0.17
Feces All Richness	Richness~ Livestock+ PropAdults+ pc1+ Elevation	0.45	0.45	Livestock	-0.003 (0.002)	-1.96	**0.05**	0.16
				Proportion Adults	-1.57 (0.67)	-2.36	**0.02**	0.23
				Climate	0.02 (0.08)	0.29	0.77	0.004
				Elevation	0.00005 (0.0001)	0.44	0.66	0.008
Saliva Vertebrate-infecting Richness	Richness~ Longitude	0.34	0.34	Longitude	-0.12 (0.04)	-3.13	**0.002**	-
Feces Vertebrate-infecting Richness	Richness~ Elevation	0.18	0.19	Elevation	0.0003 (0.0001)	-2.16	**0.03**	-

Table S9. 95% confidence set of GLMs for total saliva viral richness. Model averaging of the models presented was used to estimate effect sizes and confidence intervals for each explanatory variable. Models are ranked by ΔAICc; for each model AICc, ΔAICc, Akaike weights (*wi*) and R^2 are shown.

Model	AICc	ΔAICc	*wi*	R^2
Longitude + RawReads + 1	126.59	0	0.4	0.503
Longitude + 1	128.32	1.73	0.168	0.399
Livestock10k + Longitude + 1	129.27	2.682	0.105	0.442
Elevation + Longitude + 1	129.43	2.838	0.097	0.438
ColonyPropMales + Longitude + 1	129.54	2.951	0.091	0.436
Longitude + pc1 + 1	129.75	3.154	0.083	0.431
Longitude + OtherSppPA + 1	130.51	3.919	0.056	0.411

Table S10. 95% confidence set of GLMs for vertebrate-infecting saliva viral richness. Model averaging of the models presented was used to estimate effect sizes and confidence intervals for each explanatory variable. Models are ranked by ΔAICc; for each model AICc, ΔAICc, Akaike weights (*wi*) and R^2 are shown.

Model	AICc	delta AICc	weight	R^2
Longitude + 1	113.27	0	0.265	0.342
Longitude + pc1 + 1	113.82	0.545	0.202	0.4
Elevation + Longitude + 1	114.35	1.071	0.155	0.386
ColonyPropAdults + Longitude + 1	115.02	1.747	0.111	0.368
Livestock10k + Longitude + 1	115.27	1.994	0.098	0.361
Longitude + RawReads + 1	115.28	2.007	0.097	0.361
ColonyPropMales + Longitude + 1	115.87	2.591	0.073	0.344

Table S11. 95% confidence set of GLMs for total fecal viral richness. Model averaging of the models presented was used to estimate effect sizes and confidence intervals for each explanatory variable. Models are ranked by ΔAICc; for each model AICc, ΔAICc, Akaike weights (*wi*) and R^2 are shown.

Model	AICc	delta AICc	weight	R^2
ColonyPropAdults + Livestock10k + 1	117.02	0	0.335	0.438
ColonyPropAdults + 1	118.98	1.968	0.125	0.312
ColonyPropMales + Livestock10k + 1	120.4	3.381	0.062	0.349
ColonyPropAdults + Longitude + 1	120.75	3.734	0.052	0.338
ColonyPropAdults + OtherSppPA + 1	121.08	4.068	0.044	0.329
OtherSppPA + pc1 + 1	121.22	4.209	0.041	0.325
Livestock10k + 1	121.36	4.344	0.038	0.237
pc1 + 1	121.45	4.431	0.037	0.234
ColonyPropAdults + Fis9loc + 1	121.52	4.503	0.035	0.316
ColonyPropAdults + RawReads + 1	121.64	4.625	0.033	0.312
Elevation + 1	121.97	4.956	0.028	0.217
ColonyPropMales + pc1 + 1	122.13	5.117	0.026	0.297
ColonyPropMales + Elevation + 1	122.2	5.181	0.025	0.296
Livestock10k + Longitude + 1	122.54	5.522	0.021	0.285
Longitude + pc1 + 1	122.61	5.59	0.02	0.283
Elevation + Longitude + 1	123.1	6.08	0.016	0.267
Livestock10k + OtherSppPA + 1	123.29	6.279	0.015	0.261
ColonyPropMales + 1	123.38	6.36	0.014	0.167
Elevation + OtherSppPA + 1	123.61	6.595	0.012	0.251
Fis9loc + Livestock10k + 1	123.89	6.871	0.011	0.242
Livestock10k + RawReads + 1	124.02	7.004	0.01	0.237
ColonyPropAdults + Longitude + 1	120.75	3.734	0.052	0.338

Table S12. 95% confidence set of GLMs for vertebrate-infecting fecal viral richness. Model averaging of the models presented was used to estimate effect sizes and confidence intervals for each explanatory variable. Models are ranked by ΔAICc; for each model AICc, ΔAICc, Akaike weights (*wi*) and R^2 are shown.

Model	AICc	delta AICc	weight	R^2
Elevation + 1	78.98	0	0.161	0.18
ColonyPropAdults + 1	80.04	1.053	0.095	0.142
Elevation + OtherSppPA + 1	80.23	1.25	0.086	0.229
Elevation + Longitude + 1	80.62	1.637	0.071	0.216
pc1 + 1	80.91	1.929	0.061	0.109
OtherSppPA + pc1 + 1	81.29	2.305	0.051	0.193
Elevation + RawReads + 1	81.63	2.65	0.043	0.181
Elevation + Fis9loc + 1	81.63	2.651	0.043	0.181
ColonyPropMales + Elevation + 1	81.64	2.661	0.043	0.18
ColonyPropAdults + OtherSppPA + 1	82.01	3.023	0.036	0.167
ColonyPropAdults + Longitude + 1	82.32	3.34	0.03	0.156
ColonyPropAdults + Livestock10k + 1	82.5	3.516	0.028	0.149
ColonyPropAdults + Fis9loc + 1	82.58	3.599	0.027	0.146
ColonyPropAdults + RawReads + 1	82.63	3.642	0.026	0.145
Longitude + pc1 + 1	82.97	3.982	0.022	0.132
Livestock10k + 1	83.13	4.151	0.02	0.018
Fis9loc + 1	83.19	4.207	0.02	0.016
OtherSppPA + 1	83.36	4.373	0.018	0.009
ColonyPropMales + 1	83.37	4.391	0.018	0.008
Longitude + 1	83.48	4.501	0.017	0.003
RawReads + 1	83.52	4.535	0.017	0.002
pc1 + RawReads + 1	83.57	4.586	0.016	0.109
ColonyPropMales + pc1 + 1	83.57	4.591	0.016	0.109
Fis9loc + pc1 + 1	83.57	4.591	0.016	0.109
Livestock10k + OtherSppPA + 1	85.42	6.44	0.006	0.034
Livestock10k + Longitude + 1	85.56	6.577	0.006	0.028
ColonyPropMales + Fis9loc + 1	85.6	6.613	0.006	0.027

Table S13. Multivariate analyses of vampire bat viral community composition. PERMANOVA and GLM analyses were used to test whether variables found to impact viral richness in vampire bats also affect community composition in the same dataset. Analyses were performed separately for different sample types (feces and saliva) and virus datasets (all viruses and vertebrate-infecting viruses).

		PERMANOVA				GLM	
		d.f.	*F*	R^2	*P*-value†	LR	*P*-value†
All Virus Community Saliva	Longitude	1,22	1.61	0.07	0.14	110.1	0.08
	Raw Reads	1,22	1.38	0.06	0.18	77.41	0.18
Vertebrate-infecting Virus Community Saliva	Longitude	1,21	2.19	0.1	**0.03**	90.81	**0.005**
All Virus Community Feces	Livestock	1,22	2.193	0.095	**0.01**	142.6	**0.03**
	Proportion Adults	1,22	1.45	0.06	0.13	135.4	**0.05**
	Climate	1,22	2.94	0.12	**0.002**	171.1	**0.03**
	Elevation	1,22	2.36	0.10	**0.007**	143.1	0.06
Vertebrate-infecting Virus Community Feces	Proportion Adults	1,20	1.25	0.06	0.28	39.01	0.07
	Elevation	1,20	1.67	0.08	0.24	49.1	0.07

†P-values for multiple analyses applied to the same dataset are adjusted using the Benjamini-Hochberg false-discovery rate method, with significant values shown in bold

REFERENCES

Baillargeon, S. & Rivest, L.-P. (2007). The Rcapture Package: Loglinear Models for Capture-Recapture in R. *J Stat Softw*, 19, 1-31.

Chapman, D.H. (1951). Some properties of the hypergeometric distribution with applications to zoological censuses. *Univ Calif Public Stat*, 1, 131–160.

Chapuis, M.P. & Estoup, A. (2007). Microsatellite Null Alleles and Estimation of Population Differentiation. *Mol Biol Evol*, 24, 621–631.

Cormack, R.M. (1989). Log-Linear Models for Capture-Recapture. *Biometrics*, 45, 395.

Ellis, J.S., Gilbey, J., Armstrong, A., Balstad, T., Cauwelier, E., Cherbonnel, C., *et al.* (2011). Microsatellite standardization and evaluation of genotyping error in a large multi-partner research programme for conservation of Atlantic salmon (Salmo salar L.). *Genetica*, 139, 353–367.

Goudet, J. (1995). FSTAT (version 1.2): a computer program to calculate F-statistics. *J Hered*, 86, 485-486.

Johnson, P.C.D. & Haydon, D.T. (2008). Software for Quantifying and Simulating Microsatellite Genotyping Error. *Bioinform Biol Insights*, 1, BBI.S373.

Jombart, T. (2008). adegenet: a R package for the multivariate analysis of genetic markers. *Bioinformatics*, 24, 1403–1405.

Jombart, T. & Ahmed, I. (2011). adegenet 1.3-1: new tools for the analysis of genome-wide SNP data. *Bioinformatics*, 27, 3070–3071.

Kearse, M., Moir, R., Wilson, A., Stones-Havas, S., Cheung, M., Sturrock, S., *et al.* (2012). Geneious Basic: An integrated and extendable desktop software platform for the organization and analysis of sequence data. *Bioinformatics*, 28, 1647–1649.

Ogle, D.H. (2017). FSA: Fisheries Stock Analysis. R package version 0.8.17.

Piaggio, A.J., Johnston, J.J. & Perkins, S.L. (2008). Development of polymorphic microsatellite loci for the common vampire bat, Desmodus rotundus (Chiroptera: Phylostomidae). *Mol Ecol Resour*, 8, 440–442.

Pompanon, F., Bonin, A., Bellemain, E. & Taberlet, P. (2005). Genotyping errors: causes, consequences and solutions. *Nat Rev Genet*, 6, 847–846.

Puechmaille, S.J. (2016). The program structure does not reliably recover the correct population structure when sampling is uneven: subsampling and new estimators alleviate the problem. *Mol Ecol Resour*, 16, 608–627.

Streicker, D.G., Recuenco, S., Valderrama, W., Gomez Benavides, J., Vargas, I., Pacheco, V., *et al.* (2012). Ecological and anthropogenic drivers of rabies exposure in vampire bats: implications for transmission and control. *Proc R Soc B,* 279, 3384–3392.

Streicker, D.G., Winternitz, J.C., Satterfield, D.A., Condori-Condori, R.E., Broos, A., Tello, C., *et al.* (2016). Host-pathogen evolutionary signatures reveal dynamics and future invasions of vampire bat rabies. *Proc Natl Acad Sci USA*, 113, 10926–10931.

Taberlet, P., Waits, L. & Luikart, G. (1999). Noninvasive genetic sampling: look before you leap. *Trends Ecol Evol*, 14, 323–327.

Van Oosterhout, C., Hutchinson, W.F., Wills, D.P.M. & Shipley, P. (2004). micro-checker: software for identifying and correcting genotyping errors in microsatellite data. *Mol Ecol Notes*, 4, 535–538.

Weir, B.S. & Cockerham, C.C. (1984). Estimating F-statistics for the analysis of population structure. *Evolution*, 38, 1358-1370.
